# High-order dynamic Bayesian Network learning with hidden common causes for causal gene regulatory network

**DOI:** 10.1186/s12859-015-0823-6

**Published:** 2015-11-25

**Authors:** Leung-Yau Lo, Man-Leung Wong, Kin-Hong Lee, Kwong-Sak Leung

**Affiliations:** 1 Department of Computer Science and Engineering, The Chinese University of Hong Kong, Shatin, Hong Kong; 2 Department of Computing and Decision Sciences, Lingnan University, Tuen Mun, Hong Kong

**Keywords:** Gene regulatory network, High-order dynamic Bayesian Network, Hidden common cause, Causality inference

## Abstract

**Background:**

Inferring gene regulatory network (GRN) has been an important topic in Bioinformatics. Many computational methods infer the GRN from high-throughput expression data. Due to the presence of time delays in the regulatory relationships, High-Order Dynamic Bayesian Network (HO-DBN) is a good model of GRN. However, previous GRN inference methods assume *causal sufficiency*, i.e. no unobserved common cause. This assumption is convenient but unrealistic, because it is possible that relevant factors have not even been conceived of and therefore un-measured. Therefore an inference method that also handles hidden common cause(s) is highly desirable. Also, previous methods for discovering hidden common causes either do not handle multi-step time delays or restrict that the parents of hidden common causes are not observed genes.

**Results:**

We have developed a discrete HO-DBN learning algorithm that can infer also hidden common cause(s) from discrete time series expression data, with some assumptions on the conditional distribution, but is less restrictive than previous methods. We assume that each hidden variable has only observed variables as children and parents, with at least two children and possibly no parents. We also make the simplifying assumption that children of hidden variable(s) are not linked to each other. Moreover, our proposed algorithm can also utilize multiple short time series (not necessarily of the same length), as long time series are difficult to obtain.

**Conclusions:**

We have performed extensive experiments using synthetic data on GRNs of size up to 100, with up to 10 hidden nodes. Experiment results show that our proposed algorithm can recover the causal GRNs adequately given the incomplete data. Using the limited real expression data and small subnetworks of the YEASTRACT network, we have also demonstrated the potential of our algorithm on real data, though more time series expression data is needed.

## Background

Inferring gene regulatory network (GRN) has been an important topic in Bioinformatics, owing to the important role it plays in the functioning of the cell. In the cell, genes are *transcribed* and subsequently *translated* into proteins, some of which are *transcription factors* (TFs) which trigger or inhibit the transcription of other gene(s). The transcription and translation, however, take time and may have delays due to other reasons [1–4]. These delays have been known to affect the network stability, or cause oscillations [5–8]. Therefore, the GRN could be modeled as a directed network where each directed link is labeled with the delay, representing the regulation of a gene to a target gene.

Rather than experimentally determining the regulatory targets of each Transcription Factor (TF) in an expensive and time-consuming way, many computational methods attempt to infer the GRN from high-throughput microarray or RNA-seq gene expression data, which can measure the expression of thousands of genes at the same time, and allow time series expression data to be obtained when this is done for a number of time points.

However, to our knowledge, the previous GRN inference methods all implicitly make the assumption of *causal sufficiency*, i.e. there are no unobserved common cause, which is convenient but unrealistic. For example, miRNAs were previously not thought to take important roles in gene regulation. It is in principle impossible to be certain that all relevant factors or common causes have been measured, because factors that are not even conceived of cannot be measured. Therefore an inference method that also handles hidden common cause(s) is highly desirable.

### Gene network inference

Many GRN inference algorithms and models have been proposed, with different levels of details by labeling the edges with different information, see [9, 10] for surveys of GRN modelling and [11] for a survey on GRN inference algorithms for microarray expression data.

Some methods infer only an undirected network, for example Relevance network [12], ARACNE [13] and C3NET [14], all of which use mutual information as a measure of association between genes. Some infer a directed network, but without labeling the edges with time delays, e.g. [15]. Static Bayesian Network (BN) is sometimes used to model GRN, e.g. in [16]. But the downside of static BN is that no cycles are allowed, and no time delays are taken into account.

Some algorithms consider delay of only one time step, e.g. [17] which uses association rule mining; [18] which uses the classical Boolean network; [19] which uses Gaussian process for Bayesian inference of an Ordinary Differential Equation (ODE) model discretized in time; and DELDBN [20], which combines ODE model with local Bayesian analysis. In contrast to static BN, dynamic Bayesian Network (DBN) models the joint distribution of some random variables at different time points, and allows time delays. First-Order DBN allows time delays of only one time step, e.g. [21] and GlobalMIT [22].

On the other hand, High-Order DBN (HO-DBN) allows delays of multiple time steps. Many HO-DBN models are discrete and ignore intra-slice edges (i.e. no instantaneous effects), and are learnt by optimizing a score on candidate structure. Since BN learning is NP-hard [23], some DBN learning algorithms use heuristic or stochastic optimization such as simulated annealing, as in Banjo [24] (updated version allows multi-step delays); genetic algorithm, as in [25]; and greedy heuristic search in MMHO-DBN [26] (in case the number of parents is large, and exhaustive search is used otherwise). After [27] had shown it is possible to globally optimize Minimum Description Length (MDL) score [28] and Bayesian-Dirichlet equivalent (BDe) score [29] in polynomial time for certain BN and DBN model, the technique has been adapted to globally optimize the MIT score [30] in GlobalMIT for FO-DBN and GlobalMIT+ [31] for HO-DBN. GlobalMIT* is a heuristic and faster version of GlobalMIT+. Although for small or medium sized networks, GlobalMIT+ and GlobalMIT* could globally optimize the score in reasonable time, when the number of genes and time points increase, the practical time needed could still be long, as the order of the polynomial depends on the number of time points. Therefore, a good heuristic HO-DBN learning method that strikes a good balance between effectiveness and efficiency is still a useful complement.

On the continuous side, there are not many algorithms that handle multi-step delays, examples are TD-ARACNE [32], which is an extension of ARACNE; DD-lasso [33], which uses lasso [34]; and CLINDE [35], which extends the PC algorithm [36] with time delays.

### Hidden common cause

**Fig. 1:**
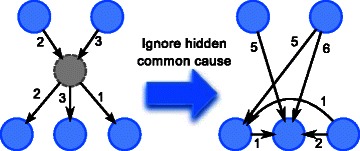
Illustration of possible misleading causal relationships if hidden common cause is ignored. The numbers are the delays. The grey circle is the hidden common cause. Since the children and parents of the hidden common cause are associated, they may be mistakenly thought to be directly linked

The above methods ignore the issue of hidden common cause(s) by (implicitly) assuming *causal sufficiency*, i.e. all common causes have been observed. Inferring hidden common cause(s) is an important topic in causality inference, because ignoring them may result in misleading causal relationships, as illustrated in Fig. 1, where some nodes are wrongly thought to be causally linked.

An early work is [37], which formulates the problem as determining the constraints on the variance-covariance matrix of observed data, and then searching for a causal structure to explain the constraints. Some works assume the presence of hidden common cause of observed variables, but only indicate that some *may* have hidden common cause, and focus on the relationships between observed variables instead. The Causal Inference (CI) and Fast Causal Inference (FCI) algorithms [36] are extensions of the PC algorithm to handle the causally insufficient case; similarly the IC* algorithm [38] is an extension of the IC algorithm. Both CI, FCI and IC* give only a partially ordered graph, where some edges may remain undirected, and some are labeled to mean the two genes may have hidden common cause. Eichler [39] is a Granger-causality based method that learns a mixed graph from time series data, where directed edges represent direct causal relationship, and dashed edges represent relationship due to hidden common cause. Pellet and Elisseeff [40] is an extension of the FCI algorithm and does not use time series data. Stochastic differential equation model (discretized in time) is used in [41], where hidden variables are assumed only to more accurately estimate the relationship between observed variables, using a convex optimization based method. In [42], a Satisfiability problem is formulated from the d-separation and d-connection information as provided by conditional tests, which is then incrementally solved to attempt to recover the dependency structure between observed variables, and some may be indicated to have latent variables, and some edges may be marked as “unknown” if the given information is insufficient for determining whether it is present or not.

While the above do not have any hidden common cause in the output, some works label predicted hidden common cause(s), but any hidden common cause can only have other hidden variables, but not observed variables as parents. Silva [43, 44] are examples in this direction, where observed variables depend linearly on its parents (either hidden or observed), and hidden variables can depend nonlinearly on its parents (only hidden variables). In [45], a linear Bayesian Network is learnt, but it is assumed that there are no edges among observed variables, and that the hidden variables are linearly independent.

Some works are less restrictive and allow the hidden variables to have observed variables as parents. Boyen et al. [46] uses a FO-DBN model, and is based on the observation that ignoring hidden variable in DBN usually results in violation of Markov property. The algorithm therefore tries to find non-markovian correlations (those across more than one time step) and try to explain them by introducing hidden variable. This work, however, evaluates the likelihood on the testing set rather than how close the resulting dependency structure is to the assumed true causal structure.

In [47], a discrete BN with hidden variables without time delays is learnt, where hidden variables are assumed to have observed variables as parents and children. It is closer to the work in this paper in that it has less restriction on the parents of the hidden common cause(s) than previously mentioned methods, but it does not handle time delays. It is motivated with the observation that the inferred dependency of the observed variables (the parents and children of the hidden variable) will usually be overly complicated, with many connections, when the hidden variable is not taken into account (see Fig. 1). Therefore, they guess the position of the hidden variable(s) and its local structure by finding semi-clique(s). A semi-clique is a subset of nodes where each node in the subset is connected to more than half of the nodes in the subset. After that adjustments are made by explicitly linking the variables of the semi-clique with the introduced hidden variable and this local structure is then fine-tuned by Structural-EM (SEM) [48]. This work also focuses on the likelihood in the evaluation instead of the inferred structure. The use of semi-clique as structural signature also places some restrictions on the subnetwork surrounding the hidden variable(s), e.g. a hidden variable must have parent(s), which are observed variable(s), and the total number of parents and children of a hidden variable must be at least three, because the smallest semi-clique has size three. Elidan and Friedman [49] complements [47] and focuses on learning the dimensionality (the number of states) of hidden variables.

In short, HO-DBN learning methods that can handle multi-step time delays such as GlobalMIT* do not handle hidden common cause(s), and hidden common cause learning algorithms do not handle multi-step time delays, and those without time delays are restricted to acyclic networks. In other words, to our knowledge, no previous methods handle multi-step time delay and learn hidden common cause(s) at the same time.

### Objective

In this paper, we want to develop a HO-DBN learning algorithm that can infer also hidden common cause(s) from discrete time series expression data, with some assumptions on the conditional distribution, but is less restrictive than the above mentioned methods. Here, we focus on the discrete case, because combinatorial regulation could be easily modeled by HO-DBN.

We assume that there is a *d*-th order (the maximum delay is *d*) stationary HO-DBN that is of interest, where a small but *unknown* number of common cause(s) are not observed. Each hidden variable has only observed variables as children and parents, with at least two children and possibly no parents. We also make the simplifying assumption that the children of unobserved variable(s) are not linked to each other, because it is difficult to differentiate whether the high association between two children are solely due to the hidden common cause, or due to both the hidden common cause and direct link between the two children. As the *prior network* is difficult to learn, and the *transition network* is of the main interest, our objective is to infer the transition network of the HO-DBN from the discrete time series of the observed variables. Moreover, it is desirable that the algorithm be capable of utilizing multiple short time series (not necessarily of the same length), as long time series are difficult to obtain. To our knowledge, previous works either make much more restrictive assumptions or ignore time delays.

## Methods

The motivating idea of our proposed method is that when a common cause is hidden, the parents of its children will not be determined correctly, and will probably be wrongly predicted to be the parent(s) of the hidden cause, or other children of the hidden cause, as illustrated in Fig. 1. In order to remedy this, the “anomaly” has to be recognized first. The overall strategy is to first learn an initial GRN while ignoring possible hidden common cause, then to detect the presence of hidden common cause(s), and estimate any detected hidden common cause. This overall strategy is similar to that of [47]. But while [47] uses semi-clique as structural signature, and [46] uses correlation across more than one time step as clue of “anomaly”, in this paper, we propose to make assumption on the conditional distribution for this purpose. The idea is that when the parents of a gene are wrongly determined, the conditional distribution will be different from expected. We could predict the genes with hidden common cause using this clue. After that, by the fact that genes with common parent should have high association, the suspected genes could be clustered, and one hidden common cause could be estimated for each cluster with size at least two. Unlike [47], we estimate hidden common cause(s) with simple EM, instead of Structural-EM.

**Fig. 2:**
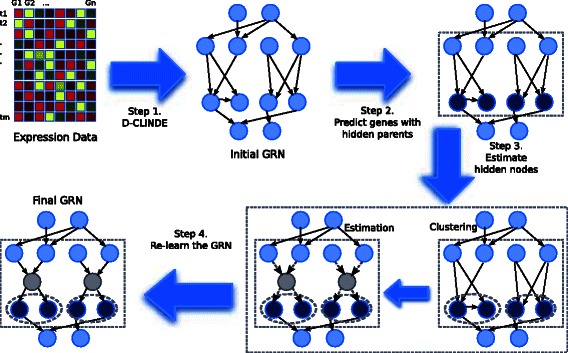
Overall Flow of the Proposed Algorithm. The steps are: 1) infer an initial GRN, 2) identify the genes with hidden common cause, 3) estimate the hidden common cause(s), which involves clustering and EM, 4) re-learn the GRN after estimation of the hidden common cause(s)

The overall flow of the proposed method is given in Fig. 2. The steps are 1) infer an initial GRN of the observed genes, 2) determine the genes with hidden common cause(s) by the estimated conditional distributions, 3) estimate the hidden common cause(s), 4) re-learn the GRN after estimation of hidden common cause(s). The program code can be obtained at https://github.com/peter19852001/hcc_dclinde. We first describe the data and model assumed in this paper, then describe each step in more details, where we first describe the case with one time series, and later we describe the case of multiple time series in a separate subsection.

### Model and data

The GRN model used in this paper is High-Order Dynamic Bayesian Network (HO-DBN) on *n*+*n*_*h*_ random variables $\textbf {X}_{t} = \{X_{1,t}, \ldots, X_{n+n_{h},t}\}\phantom {\dot {i}\!}$ at different time points *t*=1,…,*T*. Each *X*_*i,t*_ represents the expression of gene *i* at time *t*, where *n* of them are observed, and *n*_*h*_ are hidden. We assume that the DBN satisfies the *d*-th order *Markov property*:

$$\begin{array}{@{}rcl@{}} \mathrm{P}(\textbf{X}_{t} | \textbf{X}_{t-1}, \textbf{X}_{t-2}, \ldots, \textbf{X}_{1}) = \mathrm{P}(\textbf{X}_{t} | \textbf{X}_{t-1}, \ldots, \textbf{X}_{t-d}) \text{for any } t. \end{array} $$

The order *d*>1 is the maximum delay. We also assume that the DBN is stationary, i.e. the dependency P(**X**_*t*_|**X**_*t*−1_,…,**X**_*t*−*d*_) is independent of *t*. Therefore, the joint distribution can be factorized as:

(1) $$ {}\mathrm{P}(\textbf{X}_{1},\ldots,\textbf{X}_{T}) \,=\, \mathrm{P}(\textbf{X}_{1},\ldots,\textbf{X}_{d}) \prod\limits_{t=d+1}^{T}{\mathrm{P}(\textbf{X}_{t} | \textbf{X}_{t-1}, \ldots, \textbf{X}_{t-d})}  $$

The P(**X**_1_,…,**X**_*d*_) is the *prior network*, which needs many independent samples to estimate, so the focus is usually on the *transition network* P(**X**_*t*_|**X**_*t*−1_,…,**X**_*t*−*d*_). Assuming *stationary* DBN, the *transition network* could be represented as a multi-graph of *n*+*n*_*h*_ nodes (representing the *n*+*n*_*h*_ genes), where an edge *i*→*j* is labeled with a delay *τ*_*ij*_≥0, meaning that *X*_*j,t*_ depends on $X_{i,t-\tau _{\textit {ij}}}$. Note that there may be multiple edges from node *i* to node *j*, but with different delays.

We make the following assumptions on the structure of the *transition network*:

- there are *n* observed genes and *n*_*h*_ hidden variable(s).
- *n*_*h*_ is unknown, but 0≤*n*_*h*_<*n* and *n*_*h*_ is small.
- We also assume that the subgraph for which *τ*_*ij*_=0 (the *instantaneous effects* or *intra-slice edges*) is empty. This is commonly assumed, e.g. in [25], MMHO-DBN [26] and GlobalMIT+ [31]. This assumption is quite reasonable in GRN modeling, as the regulatory relationships invariably have delays. I.e. we assume that *τ*_*ij*_>0.
- each hidden variable has at least two observed genes as children.
- if a gene has a hidden parent, it has no other parents.
- children with the same hidden parent are not linked with each other.
- for each conditional distribution with *n*_*s*_ states, one of the states has probability *p*_*bias*_, and the other states each has probability of $\frac {1-p_{\textit {bias}}}{n_{s} -1}$. That is, the dependency of a gene on its parent(s) is mostly a function, with some “noise” added. A higher value of *p*_*bias*_ means a lower “noise” level.

The given data consists of *K* discrete time series $\{x_{i}^{(k)}(t): 1 \leq i \leq n, 1 \leq t \leq T_{k} \}$ with equidistant time points for 1≤*k*≤*K*. The *K* time series should be discretized in the same way, so that the states (e.g. 0, 1 and 2 for *low*, *average* and *high* expression) are consistent in different time series.

### Initial GRN

For the purpose of identifying genes with hidden common cause(s), the first step is to obtain an initial GRN. In principle, any HO-DBN learning algorithm could be used. In our preliminary test (unpublished), we have adapted CLINDE [35] to discrete data to give D-CLINDE, which is a constraint-based method extending the PC algorithm [36] (in a similar way to CLINDE). We have shown that for large number of genes and time points, D-CLINDE can be orders of magnitude faster than MMHO-DBN and GlobalMIT* (and consequently GlobalMIT+), while achieving slightly better learning performance than MMHO-DBN, and achieving 70–80 % of the learning performance of GlobalMIT*. Therefore, D-CLINDE is a good complement to GlobalMIT* (and GlobalMIT+) for large networks and time points, when even GlobalMIT* would be too slow. Also, both D-CLINDE, GlobalMIT* and GlobalMIT+ can handle multiple time series, while MMHO-DBN cannot. Therefore, in our current program, we allow the use of GlobalMIT*, GlobalMIT+ or D-CLINDE for inferring the initial GRN. We briefly describe D-CLINDE, GlobalMIT+ and GlobalMIT* in the following.

#### D-CLINDE

D-CLINDE is a constraint-based method, where conditional independence tests on the data *constrain* the possible causal structure. It consists of two stages. In stage 1, independence tests (*G* ^2^ test) are conducted on all gene pairs *i*→*j* for all possible delays up to a maximum delay. If the null hypothesis of the independence test is rejected (the score of the test is − log10(*p*-value), and the null hypothesis is rejected if the score is larger than a score threshold), the link with the associated delay is kept for stage 2. The default value for score threshold is 2, corresponding to a *p*-value threshold of 0.01. Note that there may be multiple delays for *i*→*j* after stage 1. Stage 2 attempts to eliminate indirect effects based on the fact that if *x* and *y* are conditionally independent given a set of variable(s) *Z* (not containing either *x* or *y*), then there should not be a link between *x* and *y*. So in stage 2, we iteratively condition on *h*=1 neighbor for each link to see if a link could be pruned, then condition on *h*=2 neighbors for any remaining links, and so on up to *h*=*N*_0_ for a given parameter *N*_0_, with a default value of 2. When performing a conditional test, the neighbors to be conditioned on are shifted using the delays estimated in stage 1, and if the null hypothesis is not rejected (based on score and score threshold, and is similar to stage 1), the link is pruned.

#### GlobalMIT+ and GlobalMIT*

GlobalMIT+ [31] is a method that globally maximizes the MIT score [30], which measures the mutual information between a gene and its parents (with delay), together with a penalty on the number of parents. The basic idea is that for each gene, we enumerate the parents (with the delays), starting from zero parents, then one parent, then two and so on, but with pruning to avoid having to enumerate all possible subsets.

The characteristics of the score (to be minimized) that allows effective optimization (in polynomial time) and pruning are:

- *No need to check acyclicity*: this allows the score to be calculated separately for each variable. Since GlobalMIT+ ignores *instantaneous effects*, so the network is always acyclic.
- *Additivity*: the score of a candidate network can be decomposed into the sum of the score of each gene. This greatly simplifies the search, and allows easy parallelization.
- *Splitting*: the score for each gene could be decomposed into a sum of *complexity* and *accuracy* parts as *s*(**Pa**)=*u*(**Pa**)+*v*(**Pa**), where both *u*(.)≥0 and *v*(.)≥0, and that the complexity part is “non-decreasing”: *u*(**Pa**_1_)≤*u*(**Pa**_2_) for **Pa**_1_⊆**Pa**_2_.
- *Uniformity*: the *complexity* is only a function of the number of parents, i.e. *u*(**Pa**_1_)=*u*(**Pa**_2_) whenever |**Pa**_1_|=|**Pa**_2_|.

In minimizing the score, if the *complexity* alone exceeds the best score so far, it is safe to prune the search, as adding more parents could only worsen the score. The key to the proof of polynomial time is a logarithmic bound *p* ^∗^ on the number of parents to consider (e.g. by finding a *p* ^∗^ for which *u*(**Pa**)≥*u*(*∅*) if |**Pa**|=*p* ^∗^), so that there are $\mathcal {O}((nd)^{p^{*}})$ sets of parents with delays to consider, and each could be done in $\mathcal {O}(p^{*}T)$ time, making the whole global search polynomial.

In the case of GlobalMIT+, with a simple trick the maximization is turned into minimization, and by assuming that all variables have the same number of states *k* (for *uniformity*), all of the above conditions are satisfied, so the MIT score could be optimized in polynomial time with *p* ^∗^≈ log*k*(*N*_*e*_) where *N*_*e*_ is $\sum _{i=1}^{K}{(T_{i} - d)}$. However, the order of the polynomial depends on the number of time points, and for large networks and large number of time points, the practical running time could still be long.

Recognizing this shortcoming, GlobalMIT* is a heuristic and faster version of GlobalMIT+ with the additional assumption that for each pair of genes *i*→*j*, there is only one delay, and that delay has the best MIT score. So GlobalMIT* first finds the best delay individually for each pair *i*→*j*, and need not try the delays in subsequent optimization. This substantially reduces the search space, speeding up the search greatly. However, in our preliminary test, the practical running time could still be long for large number of genes and time points.

### Identification of genes with hidden common cause

Having obtained the initial GRN of the observed genes, we can estimate the conditional distribution of each gene by maximum likelihood, and then estimate the $\hat {p}_{\textit {bias}}$ of each gene, to compare with the expected bias. In this paper, we use a simple method to estimate the bias. For each gene *g*, for each configuration *Q*_*i*_ of its parent(s) **P** **a**_*g*_, we calculate the maximum probability of the conditional distribution as max*j*P(*g*=*j*|**P** **a**_*g*_=*Q*_*i*_), and we use the median of the maximum probability over the parent configurations *Q*_*i*_’s as the estimate $\hat {p}_{\textit {bias}}$ of the bias for gene *g*.

For each gene, we compare the estimated bias $\hat {p}_{\textit {bias}}$ with the expected bias *p*_*bias*_, if $|\hat {p}_{\textit {bias}} - p_{\textit {bias}}| > \rho $ we predict the gene to have hidden common cause, where *ρ* is the tolerance with a default value of 0.05. The idea is that if a gene has no hidden common cause, we expect its parents (and delays) to be correctly determined (given sufficient data), so the estimated bias should be close to expected. On the other hand, if a gene has hidden common cause, its true parents could not be determined correctly, and we expect the estimated bias to be different from expected. Those genes determined to have hidden parents are called *candidates*.

If the number of observed genes *n* is small, we assume that the expected bias is known and given. On the other hand, when *n* is larger, by the assumption that there are only a small number of hidden variables, we could attempt to estimate the expected bias from the estimated biases of the the observed genes. We simply use the median of the estimated biases as the expected bias for this study, if it is not given. We discuss a possible alternative strategy for estimating the expected bias as future works in the conclusions.

### Estimation of hidden common cause(s)

#### Clustering the candidates

We simply output the initial GRN as the final GRN if there are no *candidates*. Otherwise, based on the fact that genes with common parent are associated, we cluster the *candidates* to determine which genes have a common parent, and also to estimate their relative delays for estimating the hidden common cause(s).

Although there are many different clustering algorithms, we found that even a simple greedy clustering algorithm works adequately from our preliminary tests. The idea is that we consider each candidate in turn, and find the cluster center that is *closest* to it, and if it is close enough, it is added to that cluster; otherwise, the candidate forms a new cluster. The steps are:

1. Let the *k* *candidates* be {*g*_1_,*g*_2_,…,*g*_*k*_}
2. Set *n*_*c*_←1, *c*_1_←*g*_1_, *τ*_1_←0, *C*_1_←{*g*_1_}
3. For *i*=2,…,*k*
  - Let $d_{i} = \text {argmax}_{1 \leq j \leq n_{c}}{d(c_{j}, g_{i})}\phantom {\dot {i}\!}$, and set *τ*_*i*_ be the associated time shift of *g*_*i*_ relative to $c_{d_{i}}$
  - If $d(c_{d_{i}}, g_{i}) \geq S_{0}$, update $C_{d_{i}} \leftarrow C_{d_{i}} \cup \{g_{i}\}$
  - Otherwise, set *n*_*c*_←*n*_*c*_+1, then set $C_{n_{c}} \leftarrow \{g_{i}\}$, $c_{n_{c}} \leftarrow g_{i}$, *τ*_*i*_←0
4. Output the *n*_*c*_ clusters {*C*_*j*_:1≤*j*≤*n*_*c*_}, and the time shifts {*τ*_*i*_:1≤*i*≤*k*}

*c*_*i*_ is the center of cluster *i*, *C*_*i*_ is cluster *i*. *τ*_*i*_ is the time shift of candidate *g*_*i*_ relative to its cluster center. *d*(*x,y*) measures the similarity of two time series *x* and *y*, here we use the maximum − log10(*p*-value) of *G* ^2^ tests of the shifted time series (shift *y* relative to *x*, from −*d* to *d*, where *d* is the maximum delay). *S*_0_ is the threshold for a series to be included in a cluster, with a default value of 2.3 (from our preliminary tests, this value seems to work well, although a value of 1.3 also seems to work adequately).

#### Estimating the hidden common cause by expectation maximization

After the clustering, we would estimate a hidden common cause (estimating its time series) for each cluster with two or more members. If no cluster has size at least two, we simply output the initial GRN as the final GRN. For each cluster with size at least two, we perform up to two rounds of EM. The first round estimates a hidden common cause (as parent) of the genes in the cluster without considering potential parents of the hidden common cause. The second round uses the estimated time series of the hidden common cause to find potential parents from all observed genes (not limited to the cluster under consideration) by picking those with high associations with the estimated hidden common cause, and re-estimate the hidden common cause treating the found (if any) potential parents as parents of the hidden common cause. But note that any identified potential parents of a hidden common cause may not be the true parents of the hidden common cause, as they are found by only considering pairwise associations but not possible indirect effects. So we still rely on the relearning of the GRN after estimating hidden common cause(s) to more accurately identify the parents of the hidden common cause(s), if any. However, we expect the identified potential parents to contain useful information for the estimation of the hidden common cause.

We use simple Expectation Maximization (EM) [50] to optimize the log-likelihood, where the states of the hidden common cause at the time points are the latent variables. Let the hidden common cause to be estimated be *h*. The number of states of *h* is either given as a parameter, or the maximum of the number of states of the children if not given. We perform two rounds of EM, each with a default of 100 iterations, and with restarts. Below we briefly describe the EM steps.

**Fig. 3:**
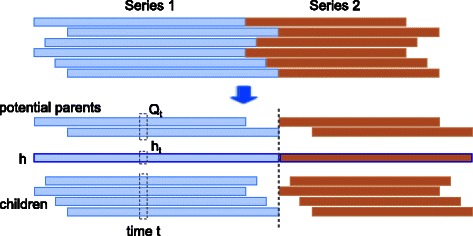
Illustration of un-aligned series for estimating hidden common cause

Suppose for cluster *C*={*g*_1_,*g*_2_,…,*g*_|*C*|_} with |*C*|>1 that we want to estimate a hidden common cause *h* with *n*_*s*_ states, which may have potential parents identified (for the second round). We first note that the different series may not be aligned because of different time shifts, as illustrated in Fig. 3. Suppose the time points of interest are *t*_*s*_≤*t*≤*t*_*e*_, we denote the state of *h* at time *t* as *h*_*t*_, which are the latent variables in the EM. Let the configuration of the potential parents of *h* be denoted by *Q*, and the value of *Q* at time *t* be denoted by *Q*_*t*_, and let *x*_*i,t*_ be the value of *g*_*i*_ at time *t* (if available). Our goal is to estimate the most probable *h*_*t*_ for *t*_*s*_≤*t*≤*t*_*e*_ given **D**={*Q*_*t*_}∪{*x*_*i,t*_}. The parameter of the likelihood is *θ*={P(*h*|*Q*)}∪{P(*g*_*i*_|*h*)}, where P(*h*|*Q*) becomes P(*h*) if *h* has no potential parents.

We first randomly initialize the parameter *θ* ^(0)^={P^(0)^(*h*|*Q*)}∪{P^(0)^(*g*_*i*_|*h*)}, then repeat the E-step and the M-step for a default of 100 iterations:

- *E-step*: at iteration *k*, for each time *t*, and for 0≤*j*<*n*_*s*_, calculate
  $$\begin{aligned} A^{(k)}_{j,t} &= \mathrm{P}(h_{t}=j, \{g_{i}=x_{i,t}\} | \theta^{(k)},\mathbf{D}) \\ &= \mathrm{P}^{(k)}(h=j|Q_{t})\prod\limits_{i}{\mathrm{P}^{(k)}(g_{i}=x_{i,t}|h=j)} \\ B^{(k)}_{j,t} &= \mathrm{P}(h_{t}=j | \theta^{(k)},\mathbf{D}) = \frac{A^{(k)}_{j,t}}{\sum_{\alpha} A^{(k)}_{\alpha,t}} \end{aligned} $$
  where *i* is over the values for which *x*_*i,t*_ has value. The log-likelihood is $L(\theta ^{(k)}) = \sum _{t} {\log \left (\sum _{j} A^{(k)}_{j,t} \right)}$. We also estimate the most probable *h*_*t*_ at iteration *k* as $h^{(k)}_{t} = \arg \max _{j} {B^{(k)}_{j,t}}$ for each *t*. If the most probable states are not changed in 3 iterations, we re-initialize *θ* randomly for the next iterations instead of performing the M-step.
- *M-step*: we update the parameter for the next iteration as follows.
  $$\begin{aligned} \mathrm{P}^{(k+1)}(h=j|Q=q) &= \frac{\sum_{t: Q_{t}=q} B^{(k)}_{j,t}}{\sum_{\alpha} \sum_{t: Q_{t}=q} B^{(k)}_{\alpha,t}} \\ \mathrm{P}^{(k+1)}(g_{i}=x|h=j) &= \frac{\sum_{t: x_{i,t}=x} B^{(k)}_{j,t}}{\sum_{\alpha} \sum_{t: x_{i,t}=\alpha} B^{(k)}_{j,t}} \\ \theta^{(k+1)} &= \{\mathrm{P}^{(k+1)}(h|Q)\} \cup \{\mathrm{P}^{(k+1)}(g_{i}|h)\} \end{aligned} $$

After the iterations, we output the $h^{(k)}_{t}$ for which *L*(*θ* ^(*k*)^) is maximum as the estimate of the most probable *h*_*t*_ for this round of EM.

After the first round, we use the estimated most probable *h*_*t*_ to find potential parents of *h*, by performing *G* ^2^ tests with all observed genes with different time shifts, using a score of − log10(*p*−*v* *a* *l* *u* *e*). A gene (with a particular time shift) could be a potential parent of *h* if the score is at least 2, and we take only 3 potential parents with the highest scores if there are more than 3. If any potential parent is found, we perform the second round of EM with the parents properly shifted to re-estimate the most probable *h*_*t*_.

Lastly, we take $h^{\prime }_{t} = h_{\alpha }$ where $\alpha = t + \max \{\max _{1 \leq k \leq |C_{i}|}{\tau _{i,k}}, d\}+1$ for 1≤*t*≤*T*−1 and $h^{\prime }_{T} = 0$ as the estimate of the hidden common cause of cluster *C*_*i*_, i.e. take the suffix of *h*_*t*_ and shift it so that $h^{\prime }_{t}$ precedes all the genes in *C* in time.

### Re-learn the GRN after estimation of hidden common cause(s)

If there are no estimated hidden common cause(s), we simply output the initial GRN as the final GRN. Otherwise we re-learn the GRN using the the original observed expression together with the estimated hidden time series of the common cause(s) to give the final GRN, but we disallow any links between the *candidates* in the same cluster. Similar to inferring the initial GRN, either GlobalMIT*, GlobalMIT+ or D-CLINDE could be used (can be chosen independently from the choice of initial GRN).

### Handling multiple time series data

**Fig. 4:**
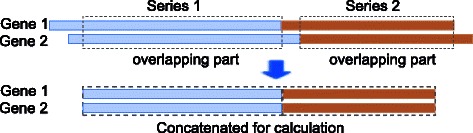
Illustration of shifting the multiple time series

The above describe the steps of the proposed algorithm when one time series data is provided, we now describe the case where multiple time series data are provided, where the series are not necessarily of the same length. The main idea is that when shifting the time series by a delay (e.g. for *G* ^2^ test), all the time series are shifted, and the overlapping parts are concatenated for the calculation. This is illustrated in Fig. 4.

Since D-CLINDE, GlobalMIT* and GlobalMIT+ can handle multiple time series, inferring the initial GRN and re-learning the GRN after estimation of hidden common cause(s) pose no difficulty.

For estimating the hidden common cause(s) using EM, for each time series, we shift according to estimated delay, and instead of only taking the overlapping parts, we “expand” each time series, and concatenate them, as illustrated in Fig. 3.

## Results and discussion

In this section, we assess the effectiveness of the proposed algorithm. Since both long time series expression of large GRN and the knowledge of true GRN are lacking, we mainly use synthetic data for evaluation. Moreover, to our knowledge, there are no previous work that infers hidden common cause(s) for HO-DBN, so we only compare our algorithm on incomplete data, with D-CLINDE and GlobalMIT* on incomplete and complete data.

We have generated three types of synthetic data for evaluation: case I) small GRN with one hidden variable and the bias is known; case II: small GRN without hidden variable and the bias is known; case III: large GRN (50 and 100 observed genes) with more than one hidden node and the bias is unknown. For each case, we generate two types of data: one long time series where we take prefixes of different lengths; and multiple short time series where we use different number of time series for different total number of time points. For cases I and II, since the networks are small, we use GlobalMIT* and D-CLINDE for inferring initial GRN and re-learning the final GRN; but for case III, since the networks are large and the number of time points required for decent performance is also large, we use only D-CLINDE to avoid long running time. In all three cases, our proposed algorithm is not given the number of hidden variables. The parameters for generating the synthetic data are summarized in Table 1, and we describe the three cases in the following sections.

**Table 1:** Parameter settings of synthetic data generation

Parameter	Case I, II	Case III
Parents (*p*)	0, 1, 2, 3	—
Children (*c*)	2, 3, 4, 5	—
Observed genes (*n*)	*p*+*c*	50, 100
Hidden nodes (*n*_*h*_)	1 for case I,	5 for *n*=50,
	II 0 for case	10 for *n*=100
*p*_*bias*_	0.65, 0.75, 0.85	0.65, 0.75, 0.85
Number of states	3	3
Maximum delay (*d*)	4	4
EM Iterations	100	1000
Replicates	20	40
Time points (*T*)	100, 200,	100, 200, 400, 800, 1000,
	400, 800	1200, 1400, 1600
Number of short time series (*K*)	4, 8, 16, 32	4, 8, 16, 32, 40, 48, 56, 64
*p*_*bias*_ known?	Yes	No

We also attempt to evaluate on real data, but as mentioned, due to the lack of long time series expression real data, it is infeasible to test our algorithm on large GRN, so we could only demonstrate our algorithm on small GRNs, but the expression data is still insufficient, so this cannot be regarded as a thorough evaluation. For this purpose, we use expression data from [51], which measures the expression of over 6,000 genes of Saccharomyces cerevisiae using DNA microarrays, with three different methods of synchronization for studying yeast cell cycle. Together with previous data from [52] (also included in [51]), there are 4 time series with information shown in Table 2. And we use YEASTRACT [53] for the GRN. YEASTRACT is a curated database of over 200,000 transcription regulatory associations in Saccharomyces cerevisiae. Since the GRN is far too large for the available expression data, we extract a small number of small subnetworks for the demonstration instead.

**Table 2:** Information of the real data time series

Series	Raw time points (Min)	Interpolated time points (Min)
alpha	every 7 mins from 0 to 119	every 10 mins from 0 to 120
cdc15	10, 30, 50, 70, 80, 90, 100,	every 10 mins from 10 to 290
	110, 120, 130, 140, 150, 160,	
	170, 180, 190, 200, 210, 220,	
	230, 240, 250, 270, 290	
cdc28	every 10 mins from 0 to 160	same time points
elu	every 30 mins from 0 to 390	every 10 mins from 0 to 390

In the following, we first describe the performance metrics, and then the generation of synthetic expression data once the GRN is given, and then describe the generation of the synthetic GRN for the different cases, and the results on the three types of synthetic data. After that, we describe the preprocessing of the YEASTRACT subnetworks and the expression data, and then present the results of our algorithm on the real data.

### Performance metrics

We assess the performance of the inference algorithm on *Links* (which is considered correct if and only if both the gene pair and the direction are correct) and *Delays* (which is considered correct if and only if both the link and the time delay *τ*_*ij*_ are correct). For each aspect, we mainly look at *F-score* as an overall measure of performance, given by *F-score* $=\frac {2*Recall*Precision}{Recall + Precision}$, where *Recall* $=\frac {TP}{TP+FN}$, *Precision* $=\frac {TP}{TP+FP}$, and *TP* is the number of *true positives*, *FP* is the number of *false positives*, *FN* is the number of *false negatives*. From our experience, usually the *Links* and *Delays* are inferred correctly at the same time, rather than getting one correct but missing the other. This is quite reasonable, as having a wrong delay may result in totally different associations, so the link is unlikely to be correct. Therefore, we focus on *Delays*, as it implies the *Links*.

**Fig. 5:**
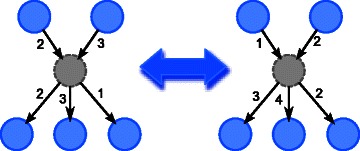
Illustration of shifting the delays for hidden variable

We still need to address the issue of comparing a predicted GRN with hidden variables against the true GRN with hidden variables, because while the hidden variables in the true GRN are labeled, the indices of the predicted hidden variable(s) may not be the same as that in the true GRN. We therefore need to map the predicted hidden variables to the true GRN before calculating the performance using the above metrics. In addition, note that for the links to/from a hidden variable, the delays cannot be completely determined. This is illustrated in Fig. 5, where the delays of links out of a hidden variable can be increased/decreased, and be compensated by the same decrease/increase in links into the hidden variable. Therefore, we may need to try different delay shifts in mapping a predicted hidden variable to true hidden variable, for useful calculation of the performance.

We try to align each predicted hidden variable to each of the true hidden nodes, and choose the one with the most matched links (to/from observed genes only) and delays (after shifting). And in case of ties, we arbitrarily choose the true hidden variable with the lowest index. After the mapping of predicted hidden variable(s), the performance of the predicted GRN is calculated as described above.

### Generation of synthetic expression data

Given a HO-DBN (the *transition network*), we generate expression data by using uniform independent distribution for the *prior network* to generate *d* (the maximum delay) time points (not included in the final expression data), then generate a time series of the required length using the conditional distributions in the *transition network*. For generating multiple short time series, the length of each series is uniformly chosen from 20 to 35.

### Case I: synthetic small GRN with one hidden node

We first test our proposed algorithm on small GRN where there is only one hidden node, and the bias *p*_*bias*_ is assumed known.

#### Network generation

**Fig. 6:**
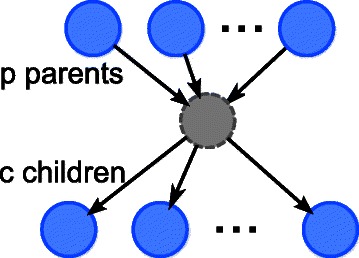
Illustration of the small synthetic network for case I. The hidden variable has *p*≥0 parents and *c*≥2 children

The GRN in this case is illustrated in Fig. 6, where there is one hidden variable, which has *p*≥0 parents and *c*≥2 children. But the algorithm is not given the number of hidden variables. For each link, the delay is uniformly chosen from {1,…,*d*}, where *d*=4. Each variable has 3 states (including the hidden variables), and the inference algorithm uses the maximum number of states of the children as the estimate of the number of states of any hidden common cause, so the predicted hidden variables also have 3 states. For each configuration of the parent(s), one state is randomly chosen as the dominant state in the conditional distribution and receives a probability of *p*_*bias*_, and the remaining states share the probability of 1−*p*_*bias*_ equally.

The different values of the parameters we have tested are shown in the column *Case I, II* of Table 1. For each setting of *p*, *c* and *p*_*bias*_, we generate 20 replicates, for a total of 960 GRNs. For the one long time series case, for each replicate, we generate expression data with 800 time points, and then take prefix to get *T* time points, and output only the expression of the observed genes. And for the multiple short time series case, for each replicate, we generate 32 time series, we test using *K* time series at a time.

#### Results

Table 3 shows the median *Delays* *F-score* of our proposed algorithm on case I with D-CLINDE and GlobalMIT* (for initial GRN and re-learning of the final GRN) using one long time series of different lengths, and Table 4 shows the results for using different number of short time series, where the medians are taken over the 20 replicates in each setting.

**Table 3:** Median delays F-scores of case I using long time series with D-CLINDE and GlobalMIT*

			*p*_*bias*_=0.65		*p*_*bias*_=0.75		*p*_*bias*_=0.85	
*p*	*c*	*T*	D-CLINDE	GlobalMIT*	D-CLINDE	GlobalMIT*	D-CLINDE	GlobalMIT*
0	2	100	0.000	0.500	0.500	0.200	0.000	0.000
		200	1.000	0.900	0.450	0.667	0.000	0.000
		400	0.900	1.000	0.000	0.000	0.000	0.000
		800	1.000	1.000	1.000	0.900	0.000	0.000
	3	100	0.400	0.400	0.400	0.400	0.367	0.400
		200	0.800	0.800	0.733	0.800	0.417	0.733
		400	0.800	0.800	0.800	0.800	0.667	0.667
		800	0.829	0.800	0.800	0.800	0.800	0.733
	4	100	0.310	0.500	0.571	0.667	0.619	0.667
		200	0.586	0.667	0.708	0.667	0.750	0.804
		400	0.667	0.708	0.750	0.857	0.675	0.708
		800	0.889	0.857	0.857	0.857	0.708	0.829
	5	100	0.444	0.500	0.667	0.708	0.667	0.667
		200	0.633	0.633	0.606	0.667	0.667	0.750
		400	0.800	0.739	0.764	0.800	0.697	0.667
		800	0.800	0.889	0.817	0.889	0.785	0.739
1	2	100	0.367	0.400	0.400	0.400	0.333	0.400
		200	0.000	0.000	0.667	0.733	0.333	0.400
		400	0.500	0.800	0.733	0.800	0.143	0.000
		800	0.733	0.800	0.733	0.800	0.619	0.733
	3	100	0.417	0.571	0.571	0.619	0.571	0.619
		200	0.500	0.571	0.750	0.857	0.708	0.536
		400	0.804	0.857	0.873	0.873	0.750	0.857
		800	0.857	0.857	0.889	0.889	0.508	0.606
	4	100	0.286	0.472	0.472	0.571	0.889	0.889
		200	0.600	0.667	0.667	0.708	0.861	1.000
		400	0.855	0.944	0.667	0.708	0.844	0.889
		800	0.909	0.909	0.800	0.817	0.800	0.889
	5	100	0.400	0.400	0.606	0.721	0.633	0.721
		200	0.633	0.600	0.769	0.800	0.692	0.785
		400	0.769	0.833	0.909	0.909	0.615	0.748
		800	0.801	0.801	0.916	0.962	0.697	0.909
2	2	100	0.268	0.310	0.333	0.571	0.000	0.000
		200	0.367	0.367	0.661	0.857	0.268	0.310
		400	0.536	0.667	0.857	0.889	0.571	0.393
		800	0.619	0.667	0.889	0.889	0.111	0.125
	3	100	0.286	0.286	0.495	0.586	0.472	0.667
		200	0.500	0.500	0.667	0.750	0.558	0.606
		400	0.422	0.500	0.708	0.775	0.718	0.750
		800	0.727	0.775	0.800	0.889	0.800	0.889
	4	100	0.348	0.500	0.500	0.667	0.472	0.667
		200	0.450	0.600	0.608	0.697	0.764	0.800
		400	0.586	0.800	0.769	0.871	0.708	0.855
		800	0.764	0.817	0.801	0.909	0.727	0.909
	5	100	0.413	0.462	0.615	0.690	0.665	0.769
		200	0.500	0.620	0.808	0.862	0.690	0.845
		400	0.742	0.833	0.857	0.878	0.857	0.923
		800	0.838	0.962	0.866	0.923	0.812	0.857
3	2	100	0.236	0.268	0.250	0.500	0.268	0.393
		200	0.222	0.250	0.500	0.571	0.286	0.619
		400	0.268	0.286	0.697	0.750	0.600	0.708
		800	0.444	0.500	0.800	0.861	0.600	0.739
	3	100	0.222	0.222	0.400	0.472	0.364	0.573
		200	0.307	0.422	0.472	0.573	0.697	0.800
		400	0.364	0.500	0.697	0.727	0.727	0.909
		800	0.600	0.727	0.727	0.800	0.801	0.909
	4	100	0.333	0.348	0.348	0.422	0.500	0.697
		200	0.382	0.473	0.552	0.667	0.813	0.833
		400	0.445	0.472	0.760	0.895	0.857	0.899
		800	0.667	0.785	0.829	0.890	0.829	0.866
	5	100	0.388	0.358	0.429	0.481	0.714	0.769
		200	0.414	0.615	0.625	0.714	0.812	0.857
		400	0.694	0.656	0.706	0.769	0.875	0.933
		800	0.708	0.866	0.789	0.857	0.904	0.933

**Table 4:** Median delays F-scores of case I using multiple short time series with D-CLINDE and GlobalMIT*

			*p*_*bias*_=0.65		*p*_*bias*_=0.75		*p*_*bias*_=0.85	
*p*	*c*	*K*	D-CLINDE	GlobalMIT*	D-CLINDE	GlobalMIT*	D-CLINDE	GlobalMIT*
0	2	4	0.000	0.000	0.000	0.250	0.000	0.000
		8	0.000	0.000	0.833	0.833	0.000	0.000
		16	0.250	0.833	1.000	1.000	0.250	0.250
		32	1.000	1.000	0.900	1.000	0.650	0.650
	3	4	0.450	0.400	0.667	0.733	0.800	0.800
		8	0.733	0.800	0.667	0.733	0.667	0.800
		16	0.667	0.800	0.800	0.800	0.667	0.667
		32	0.829	0.800	0.829	0.800	0.667	0.800
	4	4	0.536	0.619	0.619	0.762	0.762	0.857
		8	0.667	0.750	0.750	0.857	0.750	0.750
		16	0.750	0.857	0.750	0.804	0.708	0.857
		32	0.857	0.929	0.857	0.857	0.750	0.804
	5	4	0.558	0.586	0.495	0.586	0.667	0.750
		8	0.550	0.619	0.697	0.800	0.667	0.750
		16	0.800	0.764	0.889	0.899	0.633	0.739
		32	0.889	0.889	0.889	0.889	0.697	0.750
1	2	4	0.400	0.400	0.486	0.533	0.619	0.800
		8	0.452	0.533	0.667	0.800	0.619	0.733
		16	0.667	0.800	0.667	0.733	0.733	0.800
		32	0.775	0.800	0.667	0.733	0.667	0.800
	3	4	0.310	0.367	0.571	0.571	0.536	0.667
		8	0.571	0.667	0.804	0.857	0.750	0.857
		16	0.857	0.857	0.873	0.873	0.804	0.857
		32	0.857	0.857	0.829	0.873	0.829	0.873
	4	4	0.472	0.500	0.500	0.571	0.800	0.889
		8	0.500	0.536	0.667	0.775	0.899	1.000
		16	0.697	0.800	0.739	0.775	0.861	1.000
		32	0.817	0.899	0.800	0.800	0.899	0.955
	5	4	0.545	0.727	0.667	0.823	0.550	0.573
		8	0.641	0.667	0.718	0.909	0.780	0.855
		16	0.861	0.909	0.845	0.909	0.833	0.909
		32	0.899	0.909	0.857	0.916	0.769	0.883
2	2	4	0.286	0.333	0.500	0.571	0.310	0.367
		8	0.400	0.400	0.633	0.750	0.125	0.200
		16	0.571	0.667	0.750	0.873	0.250	0.333
		32	0.586	0.667	0.829	0.944	0.571	0.667
	3	4	0.286	0.393	0.495	0.571	0.472	0.536
		8	0.389	0.417	0.697	0.889	0.573	0.750
		16	0.500	0.571	0.800	0.800	0.800	0.889
		32	0.708	0.750	0.775	0.775	0.764	0.861
	4	4	0.382	0.444	0.545	0.633	0.600	0.523
		8	0.500	0.764	0.727	0.817	0.580	0.697
		16	0.748	0.785	0.833	0.909	0.801	0.871
		32	0.833	0.813	0.909	0.962	0.833	0.909
	5	4	0.333	0.431	0.571	0.718	0.678	0.688
		8	0.545	0.608	0.775	0.845	0.769	0.812
		16	0.667	0.748	0.800	0.923	0.828	0.801
		32	0.933	0.923	0.857	0.923	0.857	0.923
3	2	4	0.222	0.250	0.400	0.389	0.250	0.365
		8	0.000	0.000	0.422	0.468	0.472	0.675
		16	0.310	0.268	0.633	0.750	0.667	0.819
		32	0.472	0.500	0.727	0.800	0.667	0.750
	3	4	0.222	0.343	0.400	0.500	0.600	0.600
		8	0.422	0.500	0.472	0.600	0.606	0.697
		16	0.500	0.550	0.641	0.764	0.748	0.855
		32	0.573	0.667	0.769	0.817	0.785	0.909
	4	4	0.308	0.414	0.445	0.464	0.714	0.727
		8	0.429	0.511	0.690	0.727	0.813	0.801
		16	0.523	0.586	0.760	0.923	0.785	0.890
		32	0.690	0.739	0.800	0.923	0.857	0.899
	5	4	0.354	0.388	0.517	0.667	0.667	0.667
		8	0.517	0.615	0.607	0.746	0.789	0.857
		16	0.533	0.769	0.787	0.857	0.881	0.933
		32	0.778	0.857	0.775	0.866	0.833	0.933

First of all, we see that even for these relatively small networks, the number of time points required for decent performance is quite large. This may be due to that the algorithm does not assume that the number of hidden common cause is known. Besides, since the dependency in HO-DBN can be combinatorial (different configurations of the parents have different conditional distributions for a node), which may also be the reason that a large sample is needed.

For large *T* or *K*, our proposed algorithm can perform adequately (with either D-CLINDE or GlobalMIT*), except for *c*=2, where the performance is more erratic (e.g. *p*=2, *c*=2 and *p*_*bias*_=0.85) and may be poor even when *T*=800. One possible reason is that when *c*=2, there is less information for estimating the hidden common cause.

Also, the performance of *p*=3 is worse than the corresponding result in *p*=2. One possible reason is that with more parents, it is more difficult to identify all the *potential parents* of a hidden common cause after the first round of EM, because only pairwise association is used in the identification. Moreover, even if the *potential parents* have been correctly identified, the estimation of the hidden common cause in the second round is difficult, because there are more configurations for the parents, and consequently more conditional distributions for the hidden common cause, and therefore there are less samples in each cell of the contingency table.

Comparing using D-CLINDE and GlobalMIT* for our proposed algorithm, the difference in the performance is small when *T* or *K* is large, but usually D-CLINDE is slightly worse, which is quite reasonable because D-CLINDE is a simple heuristic.

In short, the results show that our proposed algorithm can adequately recover hidden common cause in small GRN, with large enough number of time points.

### Case II: synthetic small GRN without hidden node

We also test on small GRN without any hidden variables, where the algorithm is not given the number of hidden variables, but the bias *p*_*bias*_ is known. The parameters are the same as in case I, which are shown in the column *Case I, II* of Table 1.

#### Network generation

For each GRN (*p*, *c*, *p*_*bias*_ and replicate) in case I, we use GlobalMIT* alone on the (incomplete) data of 800 time points to infer an GRN, which is definitely wrong as all true links are to/from the hidden variable. If the inferred GRN is non-empty, it is used; otherwise, a small GRN of a node in the middle with *p* parents, and *c*−1 children is generated as in case I, but all genes are labeled as observed. Having obtained the 960 GRNs without hidden nodes, the time series are generated as in case I.

#### Results

Table 5 shows the median *Delays* *F-score* of our proposed algorithm on case II with D-CLINDE and GlobalMIT* (for initial GRN and re-learning of the final GRN) using one long time series, and Table 6 shows the corresponding results using multiple short time series.

**Table 5:** Median delays F-scores of case II using long time series with D-CLINDE and GlobalMIT*

			*p*_*bias*_=0.65		*p*_*bias*_=0.75		*p*_*bias*_=0.85	
*p*	*c*	*T*	D-CLINDE	GlobalMIT*	D-CLINDE	GlobalMIT*	D-CLINDE	GlobalMIT*
0	2	100	1.000	1.000	1.000	1.000	1.000	1.000
		200	1.000	1.000	1.000	1.000	1.000	1.000
		400	1.000	1.000	1.000	1.000	1.000	1.000
		800	1.000	1.000	1.000	1.000	1.000	1.000
	3	100	0.583	0.400	0.900	0.667	0.857	0.667
		200	0.800	1.000	0.800	1.000	1.000	1.000
		400	1.000	1.000	1.000	1.000	1.000	1.000
		800	1.000	1.000	1.000	1.000	1.000	1.000
	4	100	0.500	0.450	0.571	0.571	0.641	0.667
		200	0.733	0.889	0.708	0.889	0.718	0.906
		400	0.873	1.000	0.873	1.000	0.916	1.000
		800	0.944	1.000	0.889	1.000	0.857	1.000
	5	100	0.450	0.367	0.333	0.508	0.523	0.404
		200	0.667	0.833	0.750	0.857	0.697	0.800
		400	0.844	1.000	0.883	1.000	0.857	0.962
		800	0.889	1.000	0.916	1.000	0.857	1.000
1	2	100	0.667	0.583	0.697	0.900	0.762	0.833
		200	0.929	1.000	1.000	1.000	1.000	1.000
		400	1.000	1.000	1.000	1.000	0.733	1.000
		800	1.000	1.000	1.000	1.000	0.733	1.000
	3	100	0.417	0.472	0.571	0.686	0.733	0.775
		200	0.667	0.962	0.800	0.844	0.944	1.000
		400	0.873	1.000	0.944	1.000	1.000	1.000
		800	0.889	1.000	1.000	1.000	1.000	1.000
	4	100	0.310	0.279	0.500	0.432	0.733	0.762
		200	0.619	0.873	0.667	0.889	0.845	1.000
		400	0.800	1.000	0.899	1.000	0.899	1.000
		800	0.971	1.000	0.899	1.000	0.923	1.000
	5	100	0.333	0.321	0.369	0.414	0.400	0.422
		200	0.437	0.552	0.588	0.800	0.619	0.750
		400	0.743	0.923	0.875	1.000	0.753	0.944
		800	0.916	0.978	0.923	1.000	0.947	1.000
2	2	100	0.500	0.500	0.472	0.619	0.667	0.733
		200	0.667	0.929	0.800	1.000	0.733	1.000
		400	0.955	1.000	0.800	1.000	0.785	1.000
		800	0.889	1.000	0.889	1.000	0.829	1.000
	3	100	0.422	0.450	0.500	0.486	0.586	0.667
		200	0.667	0.829	0.800	0.889	0.667	0.857
		400	0.800	1.000	0.829	1.000	0.916	1.000
		800	0.775	1.000	0.899	1.000	0.955	1.000
	4	100	0.254	0.222	0.445	0.586	0.453	0.573
		200	0.641	0.667	0.760	0.909	0.710	0.933
		400	0.866	1.000	0.889	1.000	0.882	1.000
		800	0.899	1.000	0.916	1.000	0.889	1.000
	5	100	0.250	0.225	0.462	0.446	0.528	0.473
		200	0.400	0.500	0.633	0.857	0.703	0.769
		400	0.653	0.705	0.899	1.000	0.857	0.950
		800	0.806	0.980	0.952	0.980	0.928	1.000
3	2	100	0.250	0.111	0.500	0.500	0.558	0.857
		200	0.536	0.829	0.571	0.890	0.861	0.944
		400	0.667	0.883	0.800	1.000	0.829	1.000
		800	0.804	1.000	0.873	1.000	0.800	1.000
	3	100	0.400	0.367	0.500	0.404	0.817	0.690
		200	0.690	0.857	0.667	0.906	0.697	0.829
		400	0.800	1.000	0.775	1.000	0.857	0.944
		800	0.866	1.000	0.906	1.000	0.899	0.928
	4	100	0.310	0.250	0.464	0.602	0.444	0.591
		200	0.325	0.411	0.667	0.890	0.676	0.873
		400	0.641	0.866	0.866	1.000	0.884	0.978
		800	0.750	0.937	0.916	1.000	0.894	0.952
	5	100	0.238	0.293	0.367	0.408	0.502	0.529
		200	0.445	0.517	0.549	0.821	0.552	0.732
		400	0.646	0.823	0.814	0.958	0.781	0.923
		800	0.824	0.947	0.916	0.985	0.882	0.974

**Table 6:** Median delays F-scores of case II using multiple short time series with D-CLINDE and GlobalMIT*

			*p*_*bias*_=0.65		*p*_*bias*_=0.75		*p*_*bias*_=0.85	
*p*	*c*	*K*	D-CLINDE	GlobalMIT*	D-CLINDE	GlobalMIT*	D-CLINDE	GlobalMIT*
0	2	4	1.000	1.000	1.000	1.000	1.000	1.000
		8	1.000	1.000	1.000	1.000	1.000	1.000
		16	1.000	1.000	1.000	1.000	1.000	1.000
		32	1.000	1.000	1.000	1.000	1.000	1.000
	3	4	0.583	0.500	0.500	0.667	0.929	0.667
		8	1.000	1.000	0.900	1.000	1.000	1.000
		16	0.800	1.000	1.000	1.000	1.000	1.000
		32	0.900	1.000	1.000	1.000	1.000	1.000
	4	4	0.367	0.400	0.800	0.775	0.667	0.667
		8	0.800	0.889	0.889	1.000	0.800	0.929
		16	0.944	1.000	0.889	1.000	0.873	1.000
		32	1.000	1.000	0.889	1.000	0.857	1.000
	5	4	0.400	0.375	0.545	0.600	0.633	0.633
		8	0.750	0.873	0.750	0.889	0.785	0.909
		16	0.838	0.899	0.826	0.906	0.857	0.916
		32	0.889	1.000	0.916	1.000	0.866	0.967
1	2	4	0.667	0.667	0.800	1.000	0.800	1.000
		8	1.000	1.000	0.833	1.000	1.000	1.000
		16	0.929	1.000	1.000	1.000	1.000	1.000
		32	1.000	1.000	1.000	1.000	1.000	1.000
	3	4	0.400	0.333	0.857	0.844	0.829	0.873
		8	0.633	1.000	1.000	1.000	0.899	1.000
		16	0.889	1.000	1.000	1.000	1.000	1.000
		32	0.857	1.000	1.000	1.000	0.916	1.000
	4	4	0.367	0.292	0.500	0.750	0.619	0.829
		8	0.800	0.800	0.775	0.775	0.769	0.967
		16	0.829	1.000	0.929	1.000	0.857	1.000
		32	0.941	1.000	0.923	1.000	0.916	1.000
	5	4	0.425	0.414	0.445	0.378	0.558	0.627
		8	0.533	0.817	0.625	0.801	0.690	0.861
		16	0.703	0.940	0.812	0.933	0.817	1.000
		32	0.932	0.944	0.899	1.000	0.941	1.000
2	2	4	0.619	0.667	0.583	0.500	0.667	1.000
		8	1.000	1.000	0.708	0.929	0.733	1.000
		16	0.929	1.000	0.829	1.000	0.829	1.000
		32	1.000	1.000	1.000	1.000	0.829	1.000
	3	4	0.444	0.286	0.536	0.733	0.667	0.733
		8	0.708	0.764	0.800	0.873	0.873	1.000
		16	0.857	0.929	0.845	1.000	1.000	1.000
		32	0.857	0.967	0.873	1.000	1.000	1.000
	4	4	0.297	0.472	0.437	0.646	0.517	0.708
		8	0.523	0.667	0.733	1.000	0.800	0.921
		16	0.800	0.916	0.906	1.000	0.829	1.000
		32	0.906	0.967	0.971	1.000	0.873	1.000
	5	4	0.286	0.333	0.469	0.541	0.485	0.536
		8	0.455	0.600	0.683	0.760	0.686	0.778
		16	0.686	0.880	0.894	0.952	0.840	0.935
		32	0.777	0.935	0.952	1.000	0.928	1.000
3	2	4	0.417	0.333	0.523	0.633	0.583	0.708
		8	0.536	0.804	0.667	1.000	0.873	1.000
		16	0.750	0.906	0.882	1.000	0.873	1.000
		32	0.750	1.000	0.764	1.000	0.775	1.000
	3	4	0.364	0.310	0.472	0.785	0.500	0.697
		8	0.633	0.929	0.750	0.928	0.727	0.857
		16	0.739	1.000	0.857	1.000	0.844	0.971
		32	0.817	1.000	0.840	1.000	0.857	1.000
	4	4	0.174	0.191	0.450	0.667	0.528	0.528
		8	0.414	0.750	0.739	0.906	0.701	0.912
		16	0.558	0.857	0.909	1.000	0.781	0.950
		32	0.840	0.916	0.954	1.000	0.909	0.976
	5	4	0.216	0.195	0.401	0.400	0.505	0.574
		8	0.490	0.578	0.649	0.689	0.732	0.819
		16	0.667	0.885	0.791	0.916	0.821	0.947
		32	0.819	0.943	0.892	0.969	0.875	0.974

The performance of our algorithm using either D-CLINDE or GlobalMIT* is good when *T*≥400 or *K*≥16, and sometimes it is good even with *T*≥200 or *K*≥8. Also, in many settings, the *F-score* of using GlobalMIT* can reach 1, while D-CLINDE can sometimes reach 1. Similar to case I, using D-CLINDE is slightly worse than using GlobalMIT*.

The results show that with adequate number of time points, our proposed algorithm can infer the GRN correctly when there are no hidden common cause, and does not introduce hidden common cause needlessly.

### Case III: synthetic large GRN with more than one hidden node

Besides the above two cases for small GRN, we also test the more realistic case of larger GRN with more than one hidden node (but the number is unknown), and that the bias *p*_*bias*_ is unknown. For a network with *n* observed genes, we would generate $n_{h}=\lceil \frac {n}{10} \rceil $ hidden variables.

#### Network generation

**Fig. 7:**
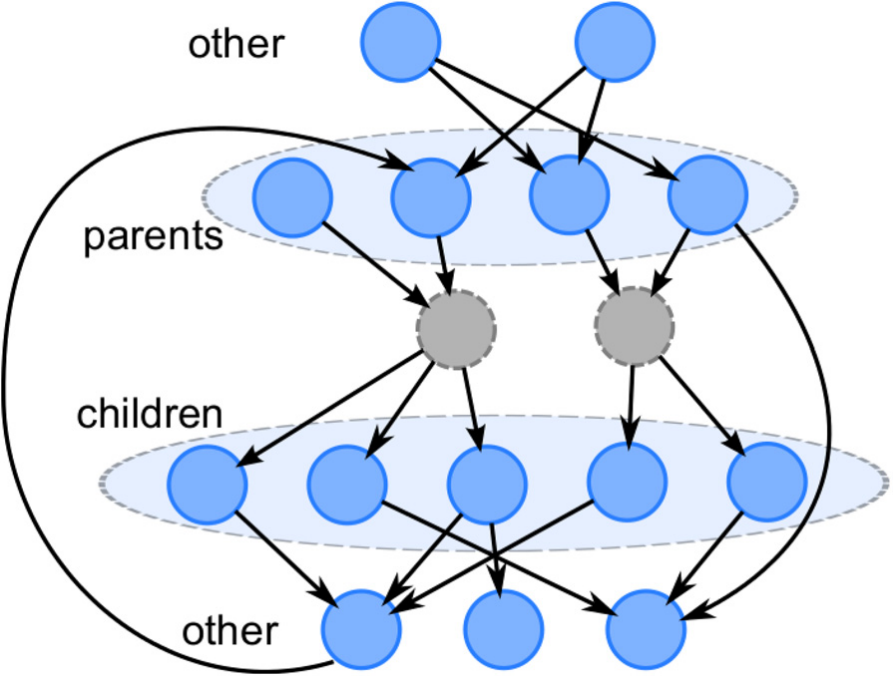
Illustration of the large synthetic network for case III. Each hidden variable has up to 3 parents, and up to 5 distinct children. The parents of hidden variables can only have *other* genes as parents, while the *other* genes can have any observed gene as parents

For *n* observed genes and *n*_*h*_ hidden nodes, a maximum of *M*_0_ parents for observed genes, a maximum of *d* as delay, we generate a GRN with the structure shown in Fig. 7, where there are four types of nodes: *hidden*, *parents of hidden*, *children of hidden*, and *other*. The hidden nodes have a random number of distinct parents and children. *Parents of hidden* take (either 1 or 2 of) *other* as parents; *other* take (either 1 or 2 of) any observed genes as parents. After generating the links, the delays and conditional distributions are generated as in cases I and II.

The parameters that we have tested are listed in column *Case III* of Table 1. For each setting of *n* and *p*_*bias*_, 40 replicates are randomly generated, for a total of 240 GRNs. For the expression data, we generate up to 1600 time points for the long time series case, and up to 64 time series for the multiple short time series case, to assess the time points needed for decent performance for networks of size 50 and 100.

#### Results

Tables 7 and 8 show the median *Delays* *F-score* on case III using one long time series and multiple short time series respectively, where *complete* is D-CLINDE alone on the complete data, which is the unrealistic case that the expression of all the *n*+*n*_*h*_ nodes are given; *hidden* is our proposed algorithm using D-CLINDE on the incomplete data, which is the more realistic case that the expression of the *n*_*h*_ hidden nodes are not given; and *ignoreHidden* is D-CLINDE alone on the incomplete data, which does not infer hidden common causes. The medians are taken over the 40 replicates in each setting. We also show the ratio of *hidden* over *complete* as percentage. We have also performed one-sided Wilcoxon signed rank tests on whether the median *F-score* of *hidden* is better than *ignoreHidden*, and show the *p*-values which are smaller than 0.1.

**Table 7:** Median delays F-scores of case III using long time series with D-CLINDE

*n*	*n*_*h*_	*p*_*bias*_	*T*	Complete (C)	Hidden (H)	IgnoreHidden	H/C	*p*-value
50	5	0.65	100	0.526	0.259	0.339	49.2 %	—
			200	0.723	0.435	0.510	60.2 %	—
			400	0.841	0.590	0.611	70.1 %	—
			800	0.898	0.757	0.647	84.3 %	6.37E-12
			1000	0.906	0.777	0.660	85.8 %	9.09E-13
			1200	0.911	0.806	0.662	88.5 %	9.09E-13
			1400	0.916	0.822	0.660	89.7 %	9.09E-13
			1600	0.923	0.839	0.660	90.9 %	9.09E-13
		0.75	100	0.669	0.356	0.455	53.1 %	—
			200	0.812	0.488	0.579	60.1 %	—
			400	0.864	0.676	0.631	78.3 %	2.20E-05
			800	0.905	0.782	0.643	86.5 %	9.09E-13
			1000	0.911	0.818	0.636	89.9 %	9.09E-13
			1200	0.910	0.828	0.629	91.0 %	1.85E-08
			1400	0.913	0.831	0.635	91.1 %	9.09E-13
			1600	0.917	0.828	0.634	90.3 %	9.09E-13
		0.85	100	0.725	0.422	0.520	58.2 %	—
			200	0.823	0.554	0.597	67.4 %	—
			400	0.884	0.702	0.634	79.5 %	1.74E-08
			800	0.911	0.803	0.641	88.1 %	9.09E-13
			1000	0.915	0.796	0.638	87.0 %	9.09E-13
			1200	0.915	0.825	0.629	90.2 %	9.09E-13
			1400	0.917	0.813	0.629	88.7 %	9.09E-13
			1600	0.912	0.818	0.625	89.7 %	9.09E-13
100	10	0.65	100	0.494	0.290	0.310	58.6 %	—
			200	0.708	0.387	0.494	54.6 %	—
			400	0.824	0.571	0.600	69.3 %	—
			800	0.883	0.715	0.639	81.0 %	2.73E-12
			1000	0.896	0.758	0.642	84.5 %	9.09E-13
			1200	0.900	0.790	0.649	87.8 %	9.09E-13
			1400	0.911	0.796	0.652	87.4 %	9.09E-13
			1600	0.913	0.801	0.646	87.8 %	9.09E-13
		0.75	100	0.647	0.362	0.442	56.0 %	—
			200	0.795	0.515	0.577	64.8 %	—
			400	0.864	0.692	0.630	80.1 %	8.22E-10
			800	0.900	0.784	0.633	87.1 %	9.09E-13
			1000	0.909	0.794	0.634	87.4 %	9.09E-13
			1200	0.916	0.805	0.638	87.9 %	9.09E-13
			1400	0.917	0.817	0.635	89.2 %	9.09E-13
			1600	0.921	0.827	0.632	89.9 %	9.09E-13
		0.85	100	0.705	0.419	0.505	59.4 %	—
			200	0.813	0.542	0.582	66.7 %	—
			400	0.883	0.690	0.624	78.1 %	1.82E-12
			800	0.912	0.766	0.627	84.1 %	9.09E-13
			1000	0.917	0.784	0.622	85.5 %	9.09E-13
			1200	0.919	0.778	0.622	84.7 %	9.09E-13
			1400	0.920	0.798	0.618	86.7 %	9.09E-13
			1600	0.926	0.790	0.616	85.3 %	9.09E-13

*Complete* is D-CLINDE on the complete data. *hidden* is our proposed algorithm with D-CLINDE on the incomplete data. *ignoreHidden* is D-CLINDE on the incomplete data. *p*-value is for one-sided Wilcoxon signed rank test on whether the median F-score of *hidden* is better than *ignoreHidden*, and entries larger than 0.1 are omitted. H/C is the ratio of *hidden* over *complete* as percentage.

**Table 8:** Median Delays F-scores of Case III using Multiple Short Time Series with D-CLINDE

*n*	*n*_*h*_	*p*_*bias*_	*K*	Complete (C)	Hidden (H)	IgnoreHidden	H/C	*p*-value
50	5	0.65	4	0.570	0.282	0.378	49.5 %	—
			8	0.745	0.426	0.533	57.2 %	—
			16	0.838	0.605	0.609	72.1 %	—
			32	0.898	0.784	0.657	87.3 %	9.09E-13
			40	0.905	0.813	0.657	89.8 %	9.09E-13
			48	0.905	0.828	0.659	91.4 %	9.09E-13
			56	0.916	0.831	0.655	90.7 %	9.09E-13
			64	0.918	0.828	0.657	90.2 %	9.09E-13
		0.75	4	0.692	0.363	0.486	52.4 %	—
			8	0.806	0.519	0.599	64.4 %	—
			16	0.871	0.708	0.638	81.2 %	1.82E-12
			32	0.912	0.786	0.640	86.2 %	9.09E-13
			40	0.917	0.826	0.641	90.1 %	9.09E-13
			48	0.919	0.834	0.636	90.8 %	9.09E-13
			56	0.920	0.853	0.636	92.7 %	9.09E-13
			64	0.918	0.847	0.626	92.3 %	9.09E-13
		0.85	4	0.740	0.429	0.535	57.9 %	—
			8	0.829	0.595	0.611	71.8 %	—
			16	0.887	0.728	0.638	82.0 %	8.00E-11
			32	0.915	0.816	0.637	89.2 %	1.82E-12
			40	0.924	0.834	0.634	90.2 %	9.09E-13
			48	0.924	0.821	0.634	88.9 %	9.09E-13
			56	0.925	0.839	0.629	90.7 %	9.09E-13
			64	0.922	0.850	0.631	92.3 %	9.09E-13
100	10	0.65	4	0.528	0.282	0.335	53.5 %	—
			8	0.725	0.417	0.509	57.6 %	—
			16	0.822	0.577	0.594	70.2 %	—
			32	0.887	0.736	0.644	83.0 %	9.09E-13
			40	0.894	0.759	0.648	84.9 %	9.09E-13
			48	0.907	0.777	0.652	85.7 %	9.09E-13
			56	0.911	0.800	0.654	87.7 %	9.09E-13
			64	0.915	0.813	0.653	88.9 %	9.09E-13
		0.75	4	0.676	0.372	0.461	55.0 %	—
			8	0.807	0.525	0.578	65.0 %	—
			16	0.873	0.680	0.630	77.9 %	6.93E-10
			32	0.910	0.784	0.648	86.2 %	9.09E-13
			40	0.915	0.813	0.642	88.9 %	9.09E-13
			48	0.917	0.828	0.643	90.3 %	9.09E-13
			56	0.922	0.822	0.642	89.2 %	9.09E-13
			64	0.923	0.836	0.635	90.6 %	9.09E-13
		0.85	4	0.731	0.428	0.517	58.5 %	—
			8	0.829	0.564	0.591	68.0 %	—
			16	0.884	0.714	0.627	80.8 %	9.09E-13
			32	0.911	0.772	0.628	84.7 %	9.09E-13
			40	0.919	0.787	0.622	85.6 %	9.09E-13
			48	0.921	0.793	0.622	86.1 %	9.09E-13
			56	0.922	0.786	0.622	85.3 %	9.09E-13
			64	0.927	0.792	0.620	85.4 %	9.09E-13

*Complete* is D-CLINDE on the complete data. *hidden* is our proposed algorithm with D-CLINDE on the incomplete data. *ignoreHidden* is D-CLINDE on the incomplete data. *p*-value is for one-sided Wilcoxon signed rank test on whether the median F-score of *hidden* is better than *ignoreHidden*, and entries larger than 0.1 are omitted. H/C is the ratio of *hidden* over *complete* as percentage.

First of all, note that *complete* can achieve good performance when *T* or *K* is large, even though D-CLINDE is only a heuristic. When *T*≥800 or *K*≥32, the performance of *complete* is better than *hidden* which in turn is better than *ignoreHidden*, which is as expected. Also, *hidden* can achieve more than 80 % of the performance of *complete*. But since having *complete* data is quite unrealistic in a real world setting, the main comparison of interest is between *hidden* and *ignoreHidden*, i.e. between handling or not handling hidden common cause. We see that *hidden* is significantly (with low *p*-value) better than *ignoreHidden* once *T*≥800 and *K*≥32.

These results show that our proposed algorithm can recover hidden common causes, for larger GRN, and where the number of hidden common causes and the bias in the conditional distributions are unknown.

### Random candidate order in clustering

By default, when clustering the candidates, they are considered sequentially from smaller index to larger index. As currently the clustering is a simple greedy algorithm, this raises the question of whether the order affects the resulting networks inferred. For this, we have added the option of using random order, and for the GRNs in case III, for each setting of *n*, *p*_*bias*_, *T* for one long time series and *K* for multiple short time series. We arbitrarily choose replicate 1 out of the 40 replicates, and repeat the inference using 100 random clustering order. Tables 9 and 10 show the mean and standard deviation of the *Links* and *Delays* *F-score* using one long time series and multiple short time series, respectively. From the results, we see that the *F-scores* of using different clustering order are very similar, and the standard deviations are all less than 0.06. This suggests that the clustering order does not have great effects on the quality of the resulting networks.

**Table 9:** Mean and standard deviation of links and delays F-scores of case III using long time series with the proposed algorithm with D-CLINDE

*n*	*n*_*h*_	*p*_*bias*_	*T*	LF mean	LF s.d.	DF mean	DF s.d.
50	5	0.65	100	0.279	0.034	0.279	0.033
			200	0.447	0.031	0.439	0.033
			400	0.597	0.026	0.595	0.025
			800	0.749	0.025	0.742	0.025
			1000	0.739	0.018	0.739	0.018
			1200	0.746	0.021	0.744	0.022
			1400	0.750	0.019	0.749	0.018
			1600	0.744	0.018	0.744	0.018
		0.75	100	0.344	0.035	0.343	0.035
			200	0.493	0.040	0.483	0.039
			400	0.734	0.047	0.732	0.047
			800	0.889	0.030	0.877	0.030
			1000	0.898	0.023	0.893	0.024
			1200	0.919	0.023	0.919	0.023
			1400	0.914	0.015	0.914	0.015
			1600	0.901	0.021	0.896	0.021
		0.85	100	0.462	0.046	0.461	0.046
			200	0.470	0.046	0.469	0.046
			400	0.755	0.053	0.755	0.053
			800	0.807	0.035	0.807	0.035
			1000	0.875	0.043	0.875	0.043
			1200	0.865	0.050	0.865	0.050
			1400	0.891	0.036	0.891	0.036
			1600	0.890	0.038	0.890	0.038
100	10	0.65	100	0.316	0.027	0.312	0.027
			200	0.400	0.025	0.398	0.025
			400	0.575	0.022	0.573	0.022
			800	0.751	0.023	0.749	0.023
			1000	0.729	0.018	0.727	0.018
			1200	0.827	0.022	0.826	0.022
			1400	0.839	0.014	0.839	0.014
			1600	0.825	0.019	0.820	0.019
		0.75	100	0.444	0.026	0.441	0.026
			200	0.569	0.027	0.567	0.028
			400	0.758	0.021	0.757	0.021
			800	0.759	0.023	0.756	0.023
			1000	0.769	0.027	0.768	0.027
			1200	0.791	0.035	0.791	0.035
			1400	0.829	0.029	0.829	0.029
			1600	0.819	0.029	0.817	0.029
		0.85	100	0.444	0.025	0.443	0.025
			200	0.503	0.031	0.502	0.030
			400	0.675	0.028	0.675	0.029
			800	0.787	0.019	0.787	0.019
			1000	0.774	0.022	0.773	0.023
			1200	0.774	0.027	0.774	0.028
			1400	0.784	0.024	0.784	0.024
			1600	0.789	0.022	0.788	0.022

The results are on the incomplete data, using replicate 1 for each setting of *n*, *p*_*bias*_ and *T*, with 100 random orders in clustering the candidates. *LF* is the *Links F-score*, and DF is the *Delays F-score*.

**Table 10:** Mean and standard deviation of links and delays F-scores of case III using multiple short time series with the proposed algorithm with D-CLINDE

*n*	*n*_*h*_	*p*_*bias*_	*K*	LF mean	LF s.d.	DF mean	DF s.d.
50	5	0.65	4	0.373	0.033	0.368	0.031
			8	0.426	0.032	0.421	0.031
			16	0.630	0.028	0.628	0.027
			32	0.740	0.019	0.734	0.018
			40	0.771	0.023	0.763	0.023
			48	0.763	0.027	0.760	0.026
			56	0.785	0.023	0.782	0.023
			64	0.802	0.027	0.789	0.028
		0.75	4	0.382	0.036	0.374	0.035
			8	0.689	0.029	0.683	0.029
			16	0.752	0.031	0.749	0.031
			32	0.869	0.032	0.869	0.033
			40	0.923	0.033	0.923	0.033
			48	0.898	0.032	0.898	0.032
			56	0.919	0.022	0.919	0.022
			64	0.887	0.023	0.887	0.023
		0.85	4	0.352	0.048	0.351	0.048
			8	0.499	0.051	0.498	0.050
			16	0.673	0.056	0.672	0.057
			32	0.808	0.049	0.807	0.048
			40	0.850	0.042	0.849	0.042
			48	0.832	0.041	0.831	0.040
			56	0.870	0.035	0.867	0.035
			64	0.890	0.025	0.890	0.025
100	10	0.65	4	0.312	0.029	0.309	0.029
			8	0.448	0.025	0.444	0.025
			16	0.604	0.027	0.599	0.028
			32	0.747	0.029	0.738	0.029
			40	0.789	0.025	0.784	0.025
			48	0.811	0.022	0.806	0.021
			56	0.801	0.026	0.795	0.026
			64	0.844	0.024	0.840	0.025
		0.75	4	0.365	0.022	0.362	0.022
			8	0.552	0.025	0.551	0.025
			16	0.678	0.023	0.673	0.023
			32	0.813	0.030	0.808	0.029
			40	0.848	0.022	0.848	0.022
			48	0.848	0.023	0.848	0.023
			56	0.862	0.019	0.861	0.019
			64	0.849	0.021	0.849	0.021
		0.85	4	0.462	0.031	0.460	0.031
			8	0.584	0.025	0.584	0.025
			16	0.708	0.029	0.708	0.029
			32	0.769	0.023	0.769	0.023
			40	0.833	0.031	0.829	0.031
			48	0.805	0.036	0.801	0.036
			56	0.817	0.030	0.813	0.030
			64	0.818	0.031	0.814	0.031

The results are on the incomplete data, using replicate 1 for each setting of *n*, *p*_*bias*_ and *K*, with 100 random orders in clustering the candidates. *LF* is the *Links F-score*, and DF is the *Delays F-score*.

### Different number of iterations in EM

The time series of hidden common causes are estimated using Expectation Maximization (EM) with random initial parameters and restarts, but EM may be sensitive to the initialization. In this subsection we repeat the experiment in case III using different number of EM iterations, namely 100, 200, 500, 1000, 2000 and 5000, to assess the effect of different number of EM iterations.

Tables 11 and 12 show the median *Delays* *F-score* of our proposed algorithm using D-CLINDE on case III with incomplete data using one long time series and multiple short time series respectively, where the number of EM iterations is varied. From the results, we can see that the median *F-scores* are very similar when using different number of EM iterations, suggesting that EM has effectively converged. In addition, as mentioned in the previous subsections, our algorithm on incomplete data (*hidden*) has decent performance, which suggests that EM has converged to a reasonably good (local) solution.

**Table 11:** Median delays F-scores of case III using long time series with the proposed algorithm with D-CLINDE

*n*	*n*_*h*_	*p*_*bias*_	*T*	em100	em200	em500	em1000	em2000	em5000
50	5	0.65	100	0.259	0.252	0.262	0.259	0.269	0.265
			200	0.430	0.420	0.431	0.435	0.426	0.431
			400	0.583	0.579	0.590	0.590	0.585	0.585
			800	0.753	0.758	0.752	0.757	0.759	0.750
			1000	0.774	0.787	0.781	0.777	0.772	0.780
			1200	0.801	0.791	0.799	0.806	0.811	0.805
			1400	0.815	0.824	0.823	0.822	0.825	0.822
			1600	0.831	0.843	0.837	0.839	0.833	0.835
		0.75	100	0.361	0.362	0.360	0.356	0.354	0.356
			200	0.477	0.484	0.485	0.488	0.486	0.494
			400	0.681	0.683	0.673	0.676	0.681	0.681
			800	0.789	0.803	0.787	0.782	0.795	0.785
			1000	0.809	0.818	0.820	0.818	0.818	0.821
			1200	0.821	0.816	0.830	0.828	0.820	0.831
			1400	0.827	0.835	0.830	0.831	0.834	0.832
			1600	0.824	0.830	0.835	0.828	0.829	0.828
		0.85	100	0.412	0.422	0.424	0.422	0.419	0.417
			200	0.569	0.565	0.555	0.554	0.555	0.573
			400	0.704	0.706	0.712	0.702	0.709	0.702
			800	0.806	0.805	0.803	0.803	0.801	0.807
			1000	0.794	0.795	0.798	0.796	0.789	0.795
			1200	0.818	0.820	0.819	0.825	0.822	0.820
			1400	0.824	0.822	0.822	0.813	0.819	0.822
			1600	0.821	0.827	0.813	0.818	0.826	0.821
100	10	0.65	1 00	0.291	0.277	0.282	0.290	0.283	0.285
			2 00	0.398	0.395	0.400	0.387	0.390	0.396
			4 00	0.566	0.575	0.576	0.571	0.571	0.574
			8 00	0.722	0.715	0.716	0.715	0.724	0.728
			1000	0.751	0.763	0.763	0.758	0.764	0.757
			1200	0.783	0.787	0.787	0.790	0.782	0.784
			1400	0.797	0.798	0.799	0.796	0.803	0.800
			1600	0.792	0.802	0.792	0.801	0.797	0.794
		0.75	100	0.360	0.370	0.358	0.362	0.363	0.356
			200	0.506	0.504	0.516	0.515	0.508	0.514
			400	0.688	0.690	0.689	0.692	0.689	0.700
			800	0.780	0.777	0.783	0.784	0.783	0.775
			1000	0.802	0.792	0.799	0.794	0.805	0.806
			1200	0.811	0.815	0.812	0.805	0.814	0.813
			1400	0.818	0.824	0.814	0.817	0.820	0.816
			1600	0.832	0.825	0.832	0.827	0.829	0.828
		0.85	100	0.412	0.426	0.424	0.419	0.415	0.408
			200	0.544	0.540	0.545	0.542	0.540	0.538
			400	0.695	0.689	0.690	0.690	0.691	0.692
			800	0.771	0.768	0.772	0.766	0.772	0.767
			1000	0.780	0.779	0.787	0.784	0.784	0.781
			1200	0.776	0.769	0.776	0.778	0.776	0.785
			1400	0.793	0.798	0.795	0.798	0.793	0.800
			1600	0.791	0.789	0.795	0.790	0.793	0.796

The results are on the incomplete data, with different number of iterations for the EM. em100 is using 100 EM iterations, em200 is using 200 EM iterations and so on.

**Table 12:** Median delays F-scores of case III using multiple short time series with the proposed algorithm with D-CLINDE

*n*	*n*_*h*_	*p*_*bias*_	*K*	em100	em200	em500	em1000	em2000	em5000
50	5	0.65	4	0.308	0.290	0.288	0.282	0.295	0.291
			8	0.433	0.442	0.432	0.426	0.421	0.433
			16	0.603	0.614	0.609	0.605	0.608	0.604
			32	0.784	0.780	0.785	0.784	0.792	0.779
			40	0.809	0.819	0.818	0.813	0.818	0.814
			48	0.828	0.830	0.831	0.828	0.829	0.836
			56	0.833	0.838	0.829	0.831	0.831	0.834
			64	0.840	0.833	0.834	0.828	0.837	0.837
		0.75	4	0.362	0.374	0.363	0.363	0.365	0.372
			8	0.523	0.520	0.513	0.519	0.519	0.523
			16	0.706	0.703	0.708	0.708	0.699	0.704
			32	0.790	0.785	0.796	0.786	0.793	0.790
			40	0.821	0.833	0.827	0.826	0.828	0.824
			48	0.838	0.834	0.835	0.834	0.827	0.836
			56	0.852	0.855	0.851	0.853	0.852	0.850
			64	0.851	0.852	0.855	0.847	0.851	0.856
		0.85	4	0.444	0.431	0.425	0.429	0.424	0.423
			8	0.591	0.578	0.582	0.595	0.599	0.599
			16	0.722	0.726	0.728	0.728	0.735	0.734
			32	0.801	0.810	0.806	0.816	0.812	0.810
			40	0.825	0.830	0.827	0.834	0.821	0.832
			48	0.829	0.825	0.827	0.821	0.826	0.828
			56	0.836	0.837	0.833	0.839	0.838	0.841
			64	0.848	0.844	0.844	0.850	0.842	0.846
100	10	0.65	4	0.281	0.284	0.277	0.282	0.285	0.280
			8	0.424	0.426	0.420	0.417	0.424	0.426
			16	0.567	0.574	0.571	0.577	0.575	0.578
			32	0.731	0.732	0.730	0.736	0.739	0.736
			40	0.755	0.762	0.767	0.759	0.757	0.763
			48	0.770	0.776	0.773	0.777	0.779	0.770
			56	0.797	0.797	0.805	0.800	0.801	0.794
			64	0.812	0.815	0.813	0.813	0.814	0.809
		0.75	4	0.371	0.374	0.374	0.372	0.375	0.368
			8	0.523	0.525	0.519	0.525	0.528	0.525
			16	0.682	0.685	0.680	0.680	0.684	0.681
			32	0.795	0.795	0.792	0.784	0.784	0.788
			40	0.815	0.816	0.815	0.813	0.820	0.820
			48	0.829	0.823	0.830	0.828	0.829	0.826
			56	0.823	0.819	0.826	0.822	0.821	0.831
			64	0.838	0.837	0.838	0.836	0.836	0.838
		0.85	4	0.431	0.430	0.431	0.428	0.432	0.435
			8	0.566	0.566	0.568	0.564	0.557	0.565
			16	0.701	0.709	0.706	0.714	0.705	0.708
			32	0.778	0.782	0.776	0.772	0.781	0.789
			40	0.780	0.789	0.788	0.787	0.787	0.787
			48	0.790	0.794	0.797	0.793	0.796	0.794
			56	0.777	0.785	0.786	0.786	0.788	0.789
			64	0.789	0.795	0.790	0.792	0.788	0.792

The results are on the incomplete data, with different number of iterations for the EM. em100 is using 100 EM iterations, em200 is using 200 EM iterations and so on.

### Small YEASTRACT subnetworks with real data

#### Preprocessing of subnetworks

YEASTRACT [53] (http://www.yeastract.com/formfindregulators.php) is accessed to get the regulating TFs of a list of 149 TFs using the “DNA binding and expression evidence” option. 392 links involving only 129 TFs are obtained and we use the “ORF List ⇔ Gene List” utility of YEASTRACT to convert the gene names into ORF id’s, and all 129 id’s appear in the yeast cell cycle [51] data.

For the limited data the GRN is still too large, so we have chosen 22 subnetworks with sizes and constituent TFs shown in Table 13. A TF (which has children in the subnetwork) is chosen to be the hidden variable in each subnetwork. Since the delays in the links are not known, we focus on the performance on *Links* for the demonstration.

**Table 13:** YEASTRACT Subnetworks

sn	*n*	*n*_*L*_	Hidden TF	Other TFs
sn1	4	5	MBP1	ASH1, HCM1, SWI4
sn2	5	11	GLN3	DAL80, GAT1, GCN4, UGA3
sn3	6	5	ADR1	IME1, MSN4, PIP2, STE12, USV1
sn4	6	5	ASH1	ACE2, MBP1, SWI5, TOS8, YHP1
sn5	6	6	YAP6	CBF1, CIN5, MOT3, PDR1, TUP1
sn6	6	10	MSN2	ADR1, FHL1, NRG1, SOK2, USV1
sn7	6	12	DAL80	GAT1, GLN3, STE12, SUM1, TEC1
sn8	7	6	ACE2	ASH1, FKH2, GAT1, HMS2, INO4, SFL1
sn9	7	7	MET4	ABF1, HAP4, MET28, MET32, SFP1, TYE7
sn10	7	7	MSN4	ADR1, HAL9, RAP1, ROX1, RPN4, SOK2
sn11	7	7	UME6	GAT1, GSM1, LEU3, MSN2, OAF1, SIP4
sn12	7	8	STE12	MIG2, MSN2, PDR1, PDR3, SOK2, YAP1
sn13	7	9	CIN5	FLO8, IXR1, NRG1, XBP1, YAP1, YAP6
sn14	7	11	MCM1	MET32, STE12, SWI4, SWI5, TYE7, YAP3
sn15	7	11	RAP1	FKH1, FKH2, MCM1, SFP1, STE12, SWI5
sn16	7	14	FLO8	CIN5, HCM1, HMS1, STE12, TEC1, TOS8
sn17	9	12	PDR1	HAP4, MET28, PDR3, RPN4, SFL1, SWI4, YAP5, YAP6
sn18	9	16	RPN4	HSF1, MSN2, MSN4, PDR1, PDR3, PUT3, REB1, YAP1
sn19	10	17	STE12	CBF1, HAP4, MET4, MSN2, PDR1, RAP1, ROX1, SOK2, YAP1
sn20	11	13	ABF1	DAL81, ECM22, HAP1, HMS2, MET4, MGA1, REB1, RTG3, STP1, SUM1
sn21	12	23	STE12	ASH1, FLO8, OAF1, RAP1, RFX1, SFP1, SKO1, SOK2, TEC1, TOS8, XBP1
sn22	13	38	ROX1	FHL1, HAP1, HAP4, HMS1, IXR1, MSN2, MSN4, SKN7, SKO1, STE12, XBP1, YAP1

*sn* is the subnetwork. *n* is the number of TFs in the subnetwork, *n*_*L*_ is the number of links in the subnetwork. The hidden TF is the one with expression hidden in *incomplete* setting of the experiments.

#### Preprocessing of expression data

The yeast cell cycle [51] data (http://genome-www.stanford.edu/cellcycle/) contains 4 time series: *alpha*, *cdc15*, *cdc28* and *elu*, with different lengths and time points, as shown in the second column of Table 2. We perform spline interpolation (using the spline() function in R) to the time points shown in the third column of Table 2 to make the time points equidistant. Some TFs in some series are entirely missing, and we fill in with zero. We rely on the spline interpolation to fill in the value for other missing values.

Since we are learning discrete HO-DBN, we perform quantile discretization to discretize the expression data into 3 states, and have prepared two sets for each subnetwork and each time series: *complete* which contains expression of all TFs of the subnetwork; and *incomplete* which omits the expression of the chosen hidden variable. Therefore, there are 8 expression datasets for each subnetwork.

#### Results

Since the subnetworks are not large, time is not a major concern, we use our proposed algorithm with D-CLINDE and GlobalMIT+. We test using one of *alpha*, *cdc15*, *cdc28* and *elu*, and also using all 4 series. The number of EM iterations is 1000. The maximum delay is 4. The bias *p*_*bias*_ is unknown. For D-CLINDE, we have tried the score thresholds 1, 1.3, 2, 2.3 and 3. For GlobalMIT+, we have tried the *α* values 0.9, 0.95, 0.99, 0.995 and 0.999.

Table 14 shows the best *Links* *F-score* over series used and parameters tested, where *complete* is D-CLINDE or GlobalMIT+ alone on the complete data, *hidden* is our proposed algorithm on incomplete data, and *ignoreHidden* is D-CLINDE or GlobalMIT+ alone on the incomplete data.

**Table 14:** Best links F-scores of YEASTRACT subnetworks using our proposed algorithm with D-CLINDE and GlobalMIT+

			D-CLINDE			GlobalMIT+		
sn	*n*	*n*_*L*_	Complete	Hidden	IgnoreHidden	Complete	Hidden	IgnoreHidden
sn1	4	5	0.600	0.571	0.571	0.286	0.500	0.267
sn2	5	11	0.533	0.429	0.429	0.453	0.659	0.421
sn3	6	5	0.333	0.571	0.000	0.400	0.364	0.000
sn4	6	5	0.364	0.308	0.000	0.267	0.400	0.000
sn5	6	6	0.400	0.500	0.000	0.250	0.364	0.000
sn6	6	10	0.414	0.387	0.400	0.343	0.480	0.286
sn7	6	12	0.429	0.476	0.430	0.353	0.316	0.267
sn8	7	6	0.571	0.667	0.000	0.444	0.381	0.000
sn9	7	7	0.267	0.588	0.000	0.545	0.444	0.222
sn10	7	7	0.364	0.286	0.000	0.462	0.400	0.000
sn11	7	7	0.250	0.364	0.000	0.286	0.286	0.000
sn12	7	8	0.462	0.667	0.500	0.286	0.308	0.333
sn13	7	9	0.381	0.677	0.133	0.364	0.636	0.000
sn14	7	11	0.250	0.594	0.267	0.250	0.500	0.250
sn15	7	11	0.361	0.411	0.361	0.250	0.316	0.200
sn16	7	14	0.320	0.333	0.222	0.258	0.308	0.207
sn17	9	12	0.222	0.444	0.125	0.325	0.522	0.154
sn18	9	16	0.293	0.404	0.190	0.299	0.333	0.167
sn19	10	17	0.174	0.286	0.182	0.195	0.289	0.195
sn20	11	13	0.214	0.778	0.148	0.200	0.568	0.105
sn21	12	23	0.216	0.250	0.108	0.205	0.321	0.212
sn22	13	38	0.226	0.210	0.195	0.180	0.252	0.183

*Complete* is D-CLINDE or GlobalMIT+ on the complete data. *hidden* is our proposed algorithm on the incomplete data (without the hidden node). *ignoreHidden* is D-CLINDE or GlobalMIT+ on the incomplete data.

When using D-CLINDE, *hidden* is better than *ignoreHidden* in 19 out of 22 subnetworks, has ties in 2 subnetworks, and is worse in 1. When using GlobalMIT+, *hidden* is better than *ignoreHidden* in 21 out of 22 subnetworks, and worse in 1. This shows that our proposed algorithm helps to infer more accurate GRN from limited data, because it considers the possibility of hidden common cause.

Also note that *hidden* is sometimes even better than *complete*, which is counter-intuitive. This suggests that the given data is insufficient to enable robust GRN inference even given the complete data. Another possible reason is that our algorithm makes the assumption that the children of a predicted hidden common cause are not linked to each other, which may help the GRN inference in the limited data case. If more time points are available, we would expect the situation to be more like the synthetic data case, where *complete* has slightly better performance than *hidden*.

As mentioned before, since the real data is very limited, we cannot draw strong conclusion for the YEASTRACT subnetworks, but the results suggest that our proposed algorithm has potential in helping to recover hidden common causes in real GRN, but likely more data is needed.

## Conclusions

In this paper, we have developed an algorithm to infer from expression data the *transition network* of a discrete HO-DBN which may have a small but unknown number of hidden common causes, with some assumptions on the conditional distributions in the HO-DBN. We have tested our algorithm on 3 types of synthetic data: small GRN with one hidden node, small GRN with no hidden node, and large GRN with a small but unknown number of hidden nodes. Experiment results show that our proposed algorithm can recover the causal GRN adequately given the incomplete data. Using the limited real expression data and small YEASTRACT subnetworks, we have also demonstrated the potential of our algorithm to recover hidden common causes in real data, but more time series expression data is needed.

### Future works

For future work, we intend to develop more sophisticated clustering of *candidate* genes with hidden common cause(s), instead of using the simple greedy heuristic. In addition, we intend to investigate different methods of specifying the similarity threshold *S*_0_. Currently the similarity of two time series is measured by the maximum − log10(*p*−*v* *a* *l* *u* *e*) of *G* ^2^ tests of the two shifted time series. Therefore, the threshold *S*_0_ is already related to *p*-value, and this helps to set an appropriate value for *S*_0_. In order to more formally set the threshold *S*_0_, one way would be to obtain the distribution of the similarity score when two time series are unrelated, and then a threshold could be set such that the probability of incorrectly putting two unrelated time series into the same cluster is controlled. However, obtaining the theoretical distribution may not be straightforward. Alternatively, the empirical distribution of the similarity score could be used. For example, first randomly choose two time series, then randomly permute the time points of one series, and calculate the similarity score. This could be repeated a large number of times to give an empirical distribution of the similarity score, and an appropriate threshold could be set accordingly.

Also, we intend to study how to relax the assumptions on the conditional distributions. Another issue worth pursuing is to better decide the number of states of hidden common causes, for example, the techniques in [49] could be incorporated.

Also, estimating the bias in the conditional distributions is an important part of the proposed algorithm, as we rely on this to predict the genes with hidden common causes. In this study, we use the maximum probability as the estimate of the bias for each conditional distribution (conditional on one configuration of the parent(s)), and use the median of the estimated biases as the estimate of the bias of a gene. If a gene has many configurations of parents, some cells in the contingency table may not have enough data points for proper estimation of the conditional distribution, and consequently the estimation of the bias may be affected. We use median as a simple strategy alleviate this problem, in the hope that more than half of the conditional distributions of a gene have proper estimation of bias. One possible alternative strategy is to use a Bayesian model, where there is an overall unknown parameter *p*_*bias*_ with a prior distribution; and given this parameter, for each gene and each configuration of the gene’s parent(s), the condition distribution has a (possibly different) dominate state which has probability *p*_*bias*_, and other states share the remaining 1−*p*_*bias*_ equally; and these conditional distributions produce the observed time series expression data. Under this model, we may attempt to estimate the posterior distribution of the parameter *p*_*bias*_, given the observed time series expression. This may better solve the issue of insufficient data points in some cells of the contingency table for the estimation of the bias. We intend to study this in more depth as future work.

In this study, we learn an initial GRN, estimate the hidden common cause(s), and re-learn the GRN to give the final GRN if any hidden common cause is learnt (steps 1 to 4 in Fig. 2). It would be interesting to see if iterating the steps 2 to 4 would allow more hidden nodes to be estimated. If the hidden common causes of the observed genes are estimated sufficiently well after the first iteration, the hidden common cause(s) (if any) of these hidden common causes might be estimated in the next iteration. However, this may be difficult, because we do not expect the true hidden time series of common causes to be recovered from the estimation. More realistically, the estimated time series may have discrepancy with the true time series, though may still allow the causal relationships of the hidden variable with other observed variables to be recovered adequately. Consequently, further estimating the hidden common causes of estimated hidden common causes would be more difficult. This is an interesting direction to investigate as future work.

## Availability of supporting data

The data set(s) supporting the results of this article is(are) included within the article (and its additional file(s)).

## Abbreviations

GRN: gene regulatory network
TF: transcription factor
BN: bayesian Network
ODE: ordinary differential equation
DBN: dynamic Bayesian Network
HO-DBN: high-order DBN
MDL: minimum description length
BDe: Bayesian-Dirichlet equivalent
CI: causal inference
FCI: fast causal inference
EM: expectation maximization
SEM: structural-EM
